# Identification of differentially expressed genes profiles in a combined mouse model of Parkinsonism and colitis

**DOI:** 10.1038/s41598-020-69695-4

**Published:** 2020-08-04

**Authors:** A. L. Gil-Martinez, L. Cuenca-Bermejo, A. M. Gonzalez-Cuello, C. Sanchez-Rodrigo, A. Parrado, S. Vyas, E. Fernandez-Villalba, M. T. Herrero

**Affiliations:** 1 Clinical and Experimental Neuroscience Group (NiCE), Institute for Aging Research, School of Medicine, Campus Mare Nostrum, University of Murcia, 30100 Murcia, Spain; 2 Biomedical Research Institute of Murcia (IMIB-Arrixaca), Campus of Health Sciences, University of Murcia, 30120 Murcia, Spain; 3 Institute of Biology Paris Seine, Gene Regulation and Adaptive Behaviours Team, Department of Neuroscience Paris Seine, Sorbonne Université, CNRS UMR 8246 & INSERM U1130, 9 Quai Saint Bernard, 75005 Paris, France

**Keywords:** Cellular neuroscience, Parkinson's disease

## Abstract

Different cellular mechanisms have been described as being potentially involved in the progression of neurodegeneration in Parkinson’s disease, although their role is still unclear. The present study aimed to identify in detail, through differentially expressed genes analysis by bioinformatics approaches, the molecular mechanisms triggered after a systemic insult in parkinsonian mice. To address this objective, we combined a dextran sodium sulfate (DSS)-induced ulcerative colitis experimental mice model with an acute 1-methyl-4-phenyl-1,2,3,6-tetradropyridine (MPTP) intoxication. The animals were divided into four experimental groups based on the different treatments: (i) control, (ii) DSS, (iii) MPTP and (iv) MPTP + DSS. The data obtained by microarray and functional enrichment analysis point out the implication of different molecular mechanisms depending on the experimental condition. We see, in the striatum of animals intoxicated only with DSS, dysfunction processes related to the blood. On the other hand, oxidative stress processes are more prominent at the MPTP intoxicated mice. Finally, differentially expressed genes within the MPTP + DSS show functional enrichment in inflammation and programmed cell death. Interestingly, we identify a significant synergistic negative effect of both toxins since the expression of differentially expressed genes (DEGs) related to balanced cellular homeostasis was not enough to prevent processes associated with cell death. This work provides detailed insights into the involvement of systemic inflammation, triggered after an insult in the colon, in the progression of the degeneration in Parkinsonism. In this way, we will be able to identify promising therapeutic targets that prevent the contribution of inflammatory processes in the progression of Parkinson’s disease.

## Introduction

Parkinson’s disease (PD) is the second most common age-related neurodegenerative disorder following Alzheimer’s disease. The main pathological feature of PD is the progressive loss of dopaminergic neurons in the Substantia Nigra pars compacta (SNpc), with the subsequent loss of dopamine (DA) in the striatum. Although much uncertainty still exists about the aetiology of PD, available evidence suggests the implication of numerous processes such as oxidative stress, mitochondrial dysfunction, inflammation and cell apoptosis. Specifically, the deleterious role of systemic inflammation in the onset and progression of PD is becoming evident, so that the interest in its study has increased in the last years^1^.

In this sense, it is showed that dopaminergic neurons are more vulnerable to oxidative stress and pro-inflammatory cytokines because of their low levels of intracellular glutathione concentrations^2^. Thus, a sustained systemic or brain inflammation involves activated microglia cells that secrete pro-inflammatory factors that damage neurons^3^. At the same time, damaged neurons release toxic factors that recruit more glial cells, resulting in a fatal vicious cycle.

To clarify the implication of systemic inflammation in PD, different combinations of experimental animal models have been reported. The most commonly used animal model to approach this issue is based on the systemic administration of lipopolysaccharide (LPS). Extensive research has shown that this endotoxin, from gram-negative bacteria, activates microglia and produces a progressive and cumulative loss of dopaminergic neurons over time^4^. For example, Qin et al. reported that LPS stimulates cells in the liver to produce TNF-α that is distributed in the blood to the brain to induce the synthesis of more TNF-α and, consequently, damaging dopaminergic neurons^5^. In another study, the systemic administration of LPS was combined with the induction of ulcerative colitis by the oral ingestion of dextran sulphate sodium (DSS). The results from this work showed an exacerbation of LPS-induced damage in the nigrostriatal system^6^. Furthermore, García-Domínguez and colleagues combined LPS with the 1-methyl-4-phenyl-1,2,3,6 tetrahydropyridine (MPTP)-based model of PD. The obtained data reinforces the activation of microglial related-events together with the exacerbation of the dopaminergic neurodegeneration^7^. Together with these observations, in our previous study, we showed an exacerbation of the dopaminergic neuronal death and the glial activation both in the SNpc and striatum when combining MPTP and DSS intoxications^8^.

Despite the published literature, further analyses are yet required to elucidate in depth the cellular and molecular mechanisms triggered after a systemic insult in Parkinsonian mice. In the present study, we performed microarray and bioinformatics analysis to identify differentially expressed genes (DEGs) of mice treated with MPTP and/or DSS. This work’s aim was to provide a better understanding of the effect of systemic inflammation on the development of PD.

## Material and methods

### Animals

The study was carried out on 16 three-months-old male C57BL/6J mice acquired from Charles River (Janvier, Le Genest Saint Isle, France). Animals were housed in a special room under regulated temperature (21 ± 1 °C) and 12-h light/dark cycles. The “Three R’s principle” was carefully applied in our study. All procedures related to animal maintenance, care and experimentation were conducted in accordance with the European Community Council Directive (2010/63/UE) for animals to be used in preclinical studies, and were approved by the Institutional Committee on Animal Ethics of the University of Murcia (REGA ES300305440012).

### Regimen of intoxication for DSS and MPTP

**Figure 1:**
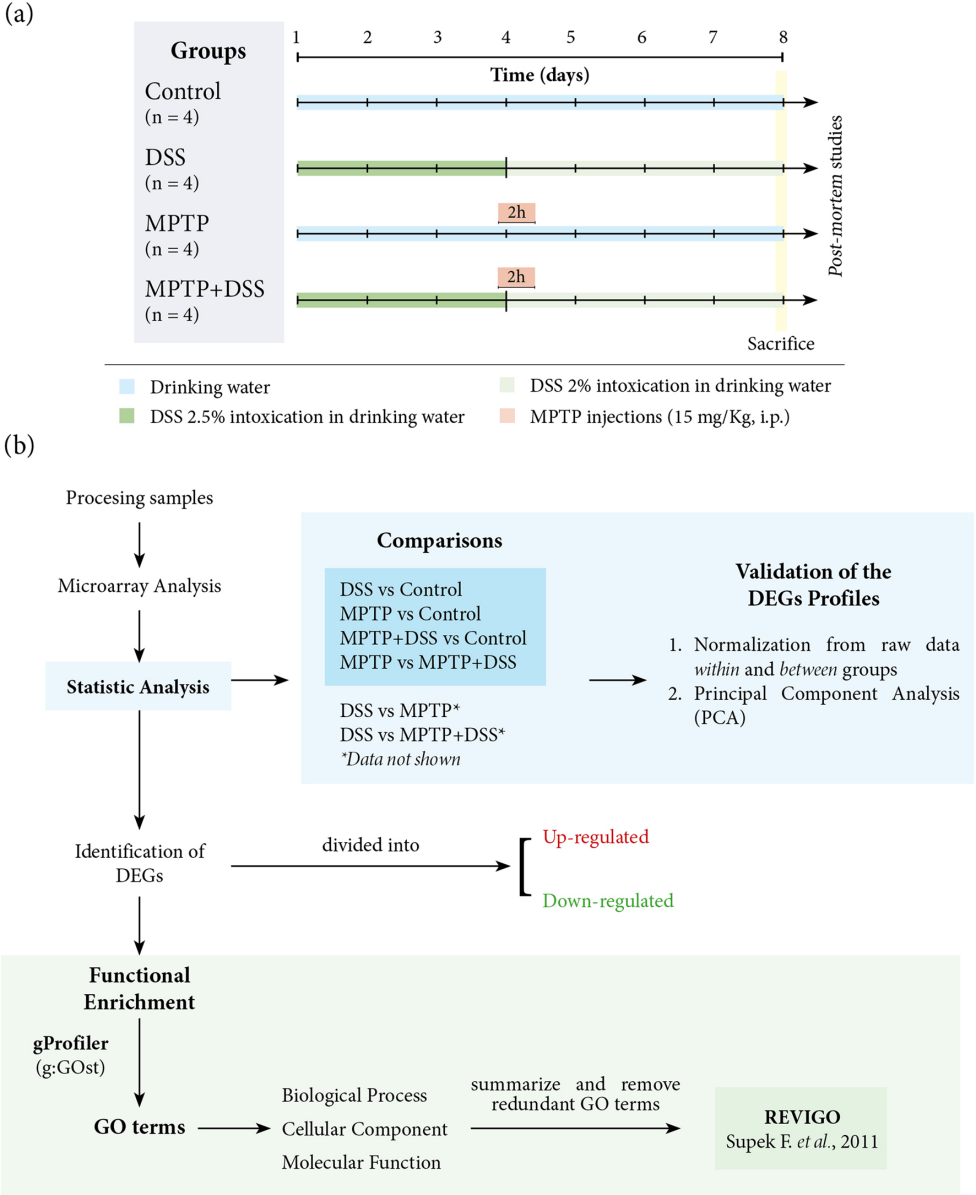
(**a**) Experimental design and mice distribution in the different groups. (**b**) Scheme of the main steps for obtaining and analyzing the results.

Ulcerative colitis intoxication was induced for 8 days by oral administration of 2–2.5% of DSS (molecular weight, 36–50 kDa, MP Biomedicals LLC, OH, USA) in tap water. On day fourth, MPTP + HCl was intraperitoneal administrated with two injections at 2-h intervals in 1 day dissolved in 0.9% saline (15 mg/Kg, Sigma Aldrich, St Quentin). Mice were distributed in four experimental groups: (a) Control (n = 4), (b) DSS (n = 4), (c) MPTP (n = 5) and (d) MPTP + DSS (n = 5) (Fig. 1a). On day 8, mice were sacrificed by decapitation and brains were immediately removed and dissected into striatum and midbrain^8^. Samples were correctly stored depending on their future use.

### RNA extraction and purification for microarray analysis

Striatums were homogenated using QIAzol from the miRNeasy Mini Kit (Qiagen, Hilden, Germany) and stored at – 80 °C until RNA extraction. Total RNA was extracted using the miRNeasy Mini Kit according to the manufacturer’s instructions. RNA samples were quantitated on a NanoDrop 2000 (Thermo Fisher Scientific, Whaltham, MA). RNA quality was examined on an Agilent 2100 Bioanalyzer (Agilent Technologies Inc., Palo Alto, CA) using the RNA 6000 Nano Kit. RNA integrity numbers (RINs) of the samples ranged between 9.2 and 9.7. Samples were stored at – 80 °C until microarray experiments.

### RNA labelling, microarray hybridization and feature extraction

RNA samples were thawed and labeled using Agilent Two Color Quick Amp Labeling and RNA Spike-In kits (Agilent), according to the manufacturer's protocol. Experimental samples were labeled with cyanine 5-CTP and used as tests. An Agilent Universal Mouse Reference RNA was labeled with cyanine 3-CTP and used as reference. Each of the labeled test cRNAs was mixed together with labeled reference cRNA. Then, mixes were hybridized onto SurePrint G3 Mouse Gene Expression v2 8 × 60 K microarrays targeting 27,122 Entrez Gene RNAs and 4,578 lncRNAs, using the Agilent Gene Expression Hybridization kit. After hybridization, the microarray slides were washed and scanned in an Agilent G2565CA DNA Microarray Scanner. Images were analyzed with the Agilent Feature Extraction software to automatically generate the datasets. Log_10_ ratios (test vs reference) were computed after normalization correction performed by linear and Lowess methods.

### Analysis of the array expression

The raw data obtained was analyzed with the statistical language R^9^ and limma package was used^10^. Intra-array normalization was performed with the method of *Loess* and inter-array normalization with the *Aquantil* method. The expression levels of the signals belonging to the same gene were grouped, and the final expression value corresponds to the mean value, as described by Limma instructions. The comparisons between the groups was: D (DSS) vs Ctrl (Control); M (MPTP) vs Ctrl; MD (MPTP + DSS) vs Ctrl; D vs M; D vs MD and M vs MD. DEGs were obtained based on the false discovery rate (FDR, < 0.05) for each comparison.

### Enrichment analysis

The enrichment analyses for the up and down DEGs were carried out with gProfileR^11^ that brings together enrichments from several databases, those of our interest were the annotations from Gene Ontology (GO) and Reactome. From the obtained GO annotations, we carefully summarize and remove redundant terms using Revigo^12^. These results were illustrated in a network diagram using Cytoscape^13^ for each ontology for comparisons: biological process (BP), molecular function (MF) and cellular component (CC) (Fig. 1b). The relationships between the nodes are based on the hierarchical association between the terms of the ontology. The brightness of the nodes refers to the level of significance.

### Ethics approval and consent to participate

Not applicable.

### Consent for publication

Not applicable.

## Results

### Differential gene expression profiles

**Figure 2:**
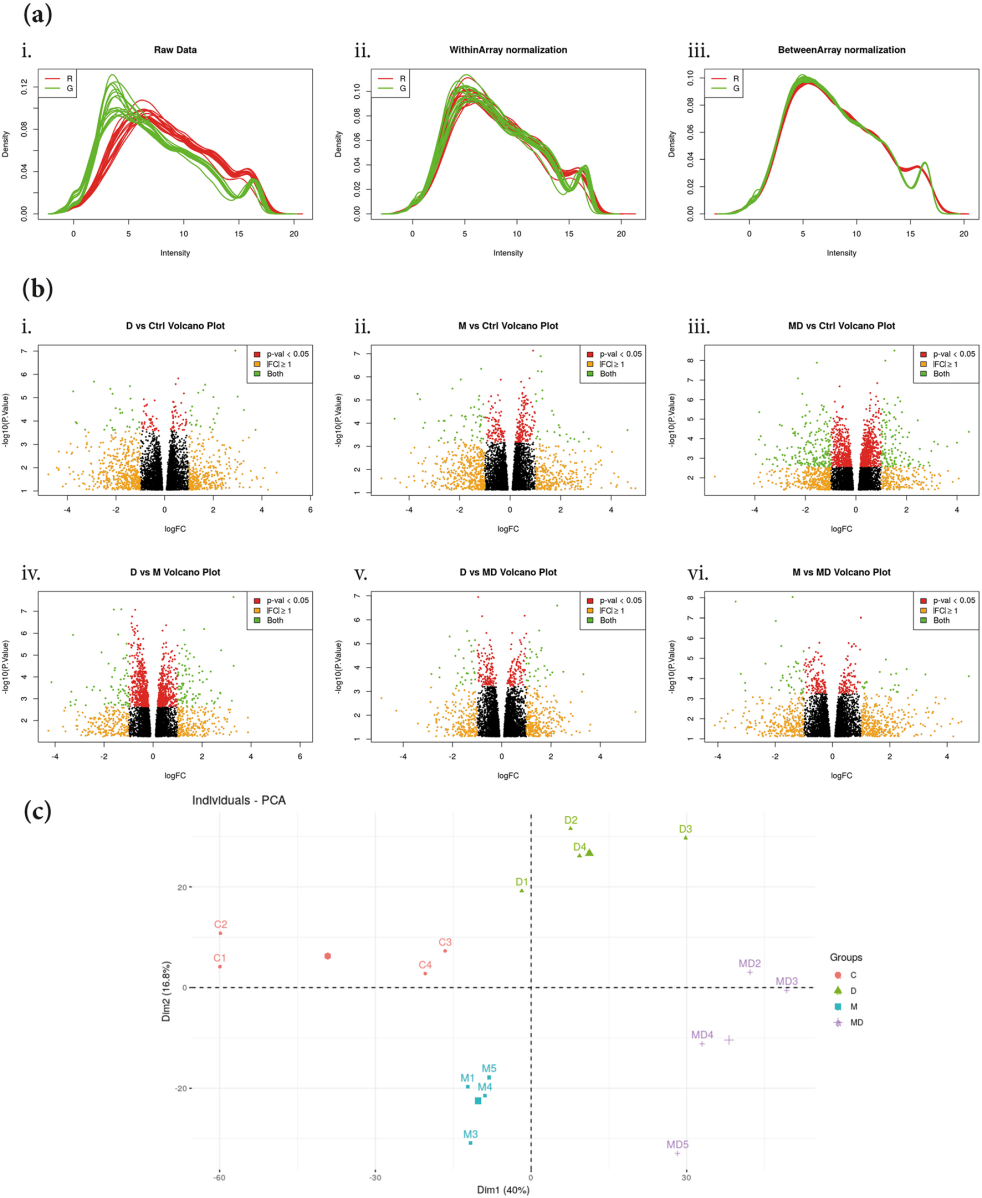
Validation of the DEGs profiles. (**a**) Normalization graphs from the raw data (i) was performed within (ii) and between (iii) groups. (**b**) Volcano plots comparing the levels of genes expressions between the experimental groups (i) DSS vs control; (ii) MPTP vs control; (iii) MPTP + DSS vs control; (iv) DSS vs MPTP; (v) DSS vs MPTP + DSS and (vi) MPTP vs MPTP + DSS. (**c**) PCA plot for the four experimental groups of interest (control, DSS, MPTP and MPTP + DSS).

Firstly, normalization from the raw data (Fig. 2a.i) was made within (Fig. 2a.ii) and between groups (Fig. 2a.iii). To avoid any supervised analysis, volcano plots from all the comparisons between the experimental groups showed the distributions of the intensities (Fig. 2b). Moreover, samples from the four experimental groups were distinctly separated in the principal component analysis (PCA) plot, indicating a differential gene expression profile caused by both intoxications (Fig. 2c).

Overall, a total of 2,413 genes were found to be differentially expressed, with an absolute fold change set at > 2 and *p*-value < 0.05 in all possible comparisons between the experimental groups [see Supplementary file 1]. Of the total DEGs, different number of genes were specifically identified based on the experimental group: DSS (132 genes), MPTP (345 genes) and MPTP + DSS (1,357 genes) compared with the control group, and MPTP compared with MPTP + DSS (281 genes) suggesting different effects in the striatum (Table 1). Moreover, in the independent hierarchical clustering represented as heat-maps, it was confirmed the DEGs within and between groups [see Supplementary file 1], specifically exacerbated in the comparisons between MPTP + DSS and control groups.

**Table 1:** Total number of DEGs in the different experimental groups [see Supplementary file 2].

Comparisons	Total DEGs	Down-regulated	Up-regulated
DSS vs ctrl	132	63	69
MPTP vs ctrl	345	150	195
MPTP + DSS vs ctrl	1,357	634	723
MPTP vs MPTP + DSS	281	124	157

### GO enrichment analysis

Based on the high amount of differential expressed genes between groups, we aimed to perform a functional enrichment analysis using g:GOSt from gProfiler which provides the most enriched GO terms together with other data sources (data shown in the Supplementary Files) associated with our gene lists.

Specifically, the data from the GO enrichment analysis is organized into three categories: (i) biological process, (ii) cellular component and (iii) molecular function. All the GO terms lists obtained in each category were summarize removing redundant terms using REVIGO. The output data was used to generate the GO-terms network graphs in Cytoscape. The setting threshold for the false discovery rate (FDR at 0.05).

### Biological process

**Figure 3:**
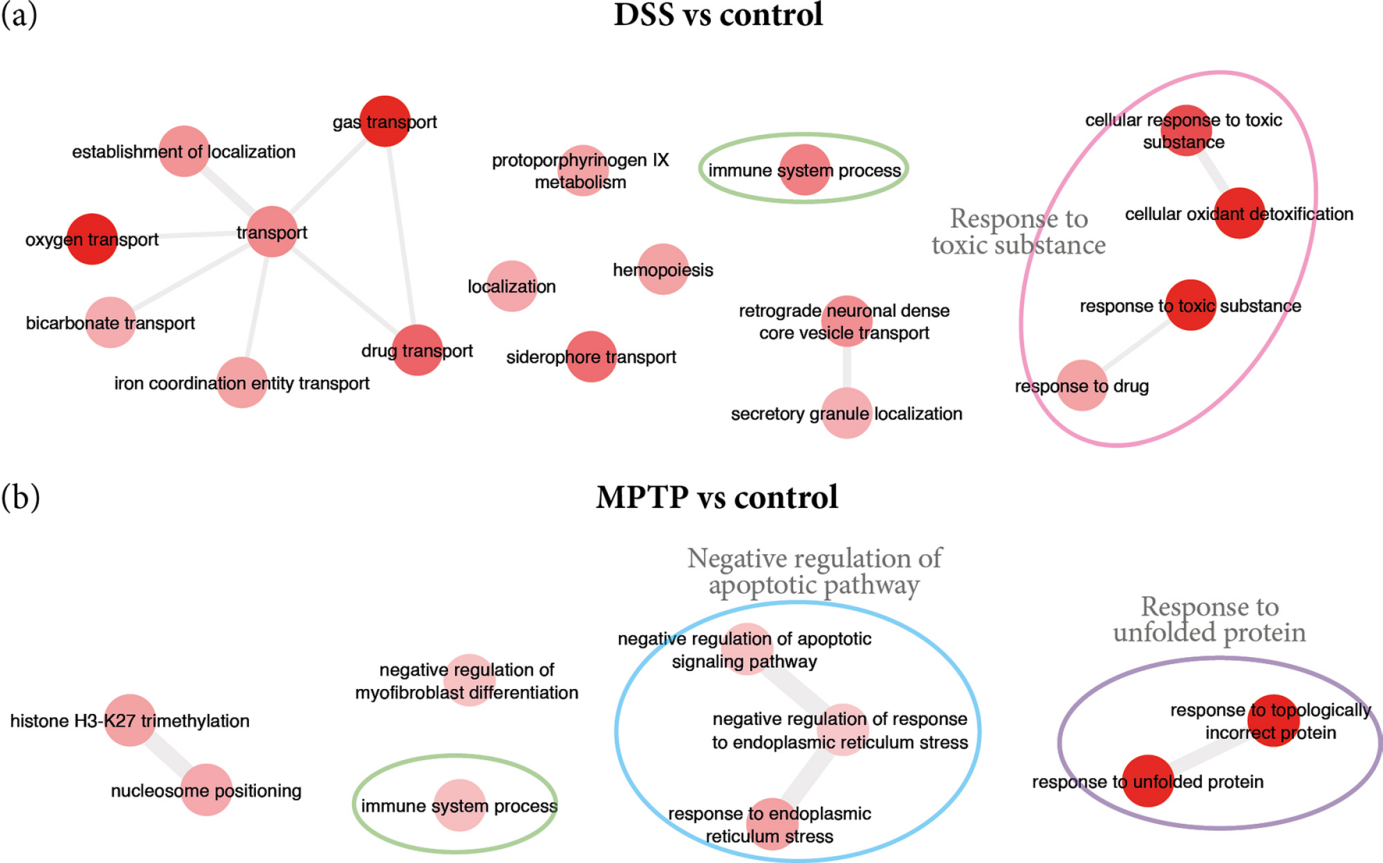
Summary of the main GO terms found for biological process in the comparisons for (**a**) DSS vs control and (**b**) MPTP vs control. The color of the circles help to better identify the groups of terms for specific processes: green for immune system process, pink for response to toxic substance, blue for apoptotic pathway and purple for response to unfolded protein.

**Figure 4:**
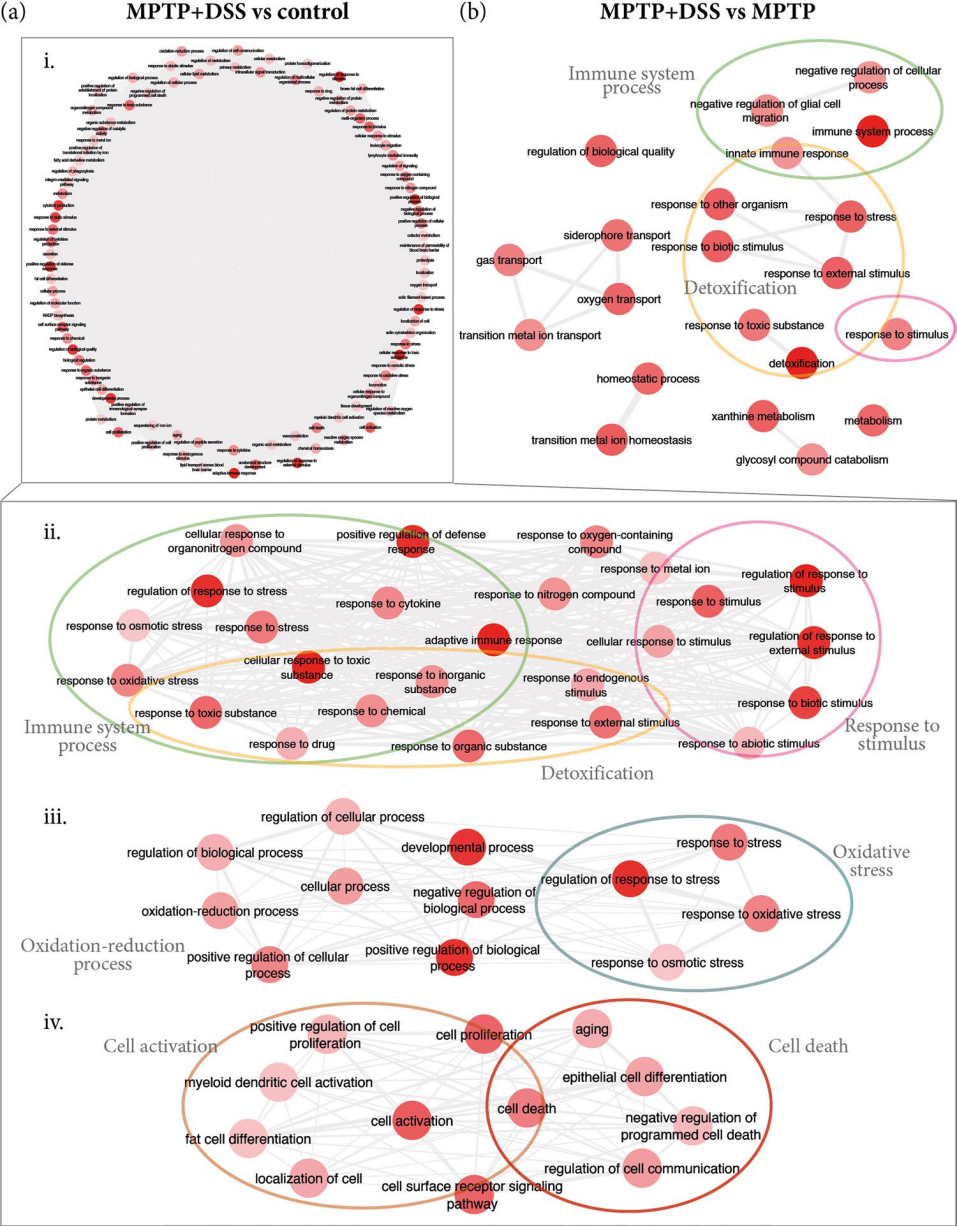
Summary of the main GO terms found for biological process in the comparisons for MPTP + DSS vs control (**a**) and MPTP (**b**, i–iv). The color of the circles help to better identify the groups of terms for specific processes: green for immune system process (ii, **b**), pink for response to toxic substance/stimulus (ii, **b**), aquamarine for oxidative stress (iii), orange for cell activation (iv) and red for cell death (iv).

Gene ontology enrichment analysis revealed different profiles depending on the experimental groups. Specifically, the results that concern biological process mainly involved GO terms for response to toxic substance/stimulus, detoxification and immune system but in different way depending on the experimental groups. Thus, up-regulated DEGs, related to response to toxic substance/stimulus, were found in groups treated with DSS (DSS and MPTP vs control; and, MPTP + DSS vs MPTP, Figs. 3 and 4). Interestingly, we found up-regulated DEGs related to detoxification mechanisms in animals treated with DSS compared to control mice (Table 2, Fig. 3a) and MPTP + DSS, clearly observed when comparing MPTP + DSS vs MPTP (Table 3, Fig. 4).

**Table 2:** The enriched GO terms for the up-regulated DEGs of DSS and MPTP vs control, respectively.

GO Term	Description	Corrected *p*-value	DEGs count
**DSS vs control**			
GO:0098869	Cellular oxidant detoxification	1.08E−04	6
GO:1990748	Cellular detoxification	1.10E−04	6
GO:0098754	Detoxification	1.44E−04	6
GO:0097237	Cellular response to toxic substance	1.00E−03	7
GO:0015893	Drug transport	2.71E−03	6
GO:0002376	Immune system process	8.49E−03	17
GO:0042493	Response to drug	3.23E−02	10
GO:0030097	Hemopoiesis	3.23E−02	9
GO:0046501	Protoporphyrinogen IX metabolic process	3.23E−02	2
GO:0048534	Hematopoietic or lymphoid organ development	4.23E−02	9
**MPTP vs control**			
GO:0035966	Response to topologically incorrect protein	7.61E−09	11
GO:0006986	Response to unfolded protein	1.67E−06	10
GO:0035967	Cellular response to topologically incorrect protein	6.57E−05	8
GO:0034620	Cellular response to unfolded protein	2.64E−04	7
GO:0030968	Endoplasmic reticulum unfolded protein response	1.55E−03	6
GO:0034976	Response to endoplasmic reticulum stress	6.22E−03	9
GO:0002376	Immune system process	2.88E−02	31
GO:2001234	Negative regulation of apoptotic signaling pathway	3.29E−02	8
GO:1904761	Negative regulation of myofibroblast differentiation	3.31E−02	2
GO:1903573	Negative regulation of response to endoplasmic reticulum stress	4.80E−02	4

**Table 3:** The enriched GO terms for the up-regulated DEGs of MPTP + DSS vs control and MPTP, respectively.

GO Term	Description	Corrected *p*-value	DEGs count
**MPTP + DSS vs control**^a^			
GO:0070887	Cellular response to chemical stimulus	3.70E−13	155
GO:0098754	Detoxification	3.36E−11	24
GO:0002376	Immune system process	5.01E−11	135
GO:0006950	Response to stress	6.10E−11	173
GO:1990748	Cellular detoxification	1.07E−09	21
GO:0002682	Regulation of immune system process	1.65E−09	86
GO:0009605	Response to external stimulus	3.46E−09	135
GO:0006954	Inflammatory response	5.40E−09	53
GO:0050896	Response to stimulus	2.14E−08	345
GO:0097237	Cellular response to toxic substance	3.37E−08	29
GO:0080134	Regulation of response to stress	3.37E−08	78
GO:0009636	Response to toxic substance	3.37E−08	45
GO:0042127	Regulation of cell population proliferation	4.34E−08	93
GO:0006952	Defense response	4.34E−08	88
GO:0031347	Regulation of defense response	4.34E−08	48
GO:0098869	Cellular oxidant detoxification	4.52E−08	18
GO:0042592	Homeostatic process	7.16E−08	98
GO:0032502	Developmental process	1.14E−07	238
GO:0032101	Regulation of response to external stimulus	1.14E−07	60
GO:0001775	Cell activation	1.39E−07	65
GO:0048583	Regulation of response to stimulus	6.53E−07	167
GO:0001816	Cytokine production	8.61E−07	49
GO:0048856	Anatomical structure development	9.28E−07	220
**MPTP + DSS vs MPTP**^b^			
GO:0098754	Detoxification	5.56E−04	8
GO:0002376	Immune system process	5.56E−04	34
GO:1990748	Cellular detoxification	2.93E−03	7
GO:0055076	Transition metal ion homeostasis	8.11E−03	7
GO:0097577	Sequestering of iron ion	9.03E−03	2
GO:0051707	Response to other organism	9.03E−03	21
GO:0046916	Cellular transition metal ion homeostasis	9.03E−03	6
GO:0046110	Xanthine metabolic process	9.03E−03	2
GO:0098869	Cellular oxidant detoxification	9.03E−03	6
GO:0043207	Response to external biotic stimulus	9.03E−03	21
GO:0009607	Response to biotic stimulus	9.03E−03	21
GO:0009605	Response to external stimulus	9.44E−03	32

See complete tables in the Supplementary Files.

^a^Results shown for MPTP + DSS vs control with corrected *p*-value ≤ 1E-07.

^b^Results shown for MPTP + DSS vs MPTP with corrected *p*-value ≤ 1E-03.

Moreover, the data revealed that genes related to immune system processes are up-regulated in DSS (Fig. 3a), MPTP (Fig. 3b) and MPTP + DSS (Fig. 4a) groups compared to the control group; and, when comparing MPTP + DSS with MPTP group (Fig. 4b). However, these genes are especially exacerbated when comparing MPTP + DSS with control and MPTP mice (Fig. 4a.ii and Table 3). Together with the exacerbation of the immune system process, we also observed in this group oxidative stress processes (Fig. 4a.iii and Table 3), cell activation and cell death (Fig. 4a.iii and Table 3).

### Cellular component

**Figure 5:**
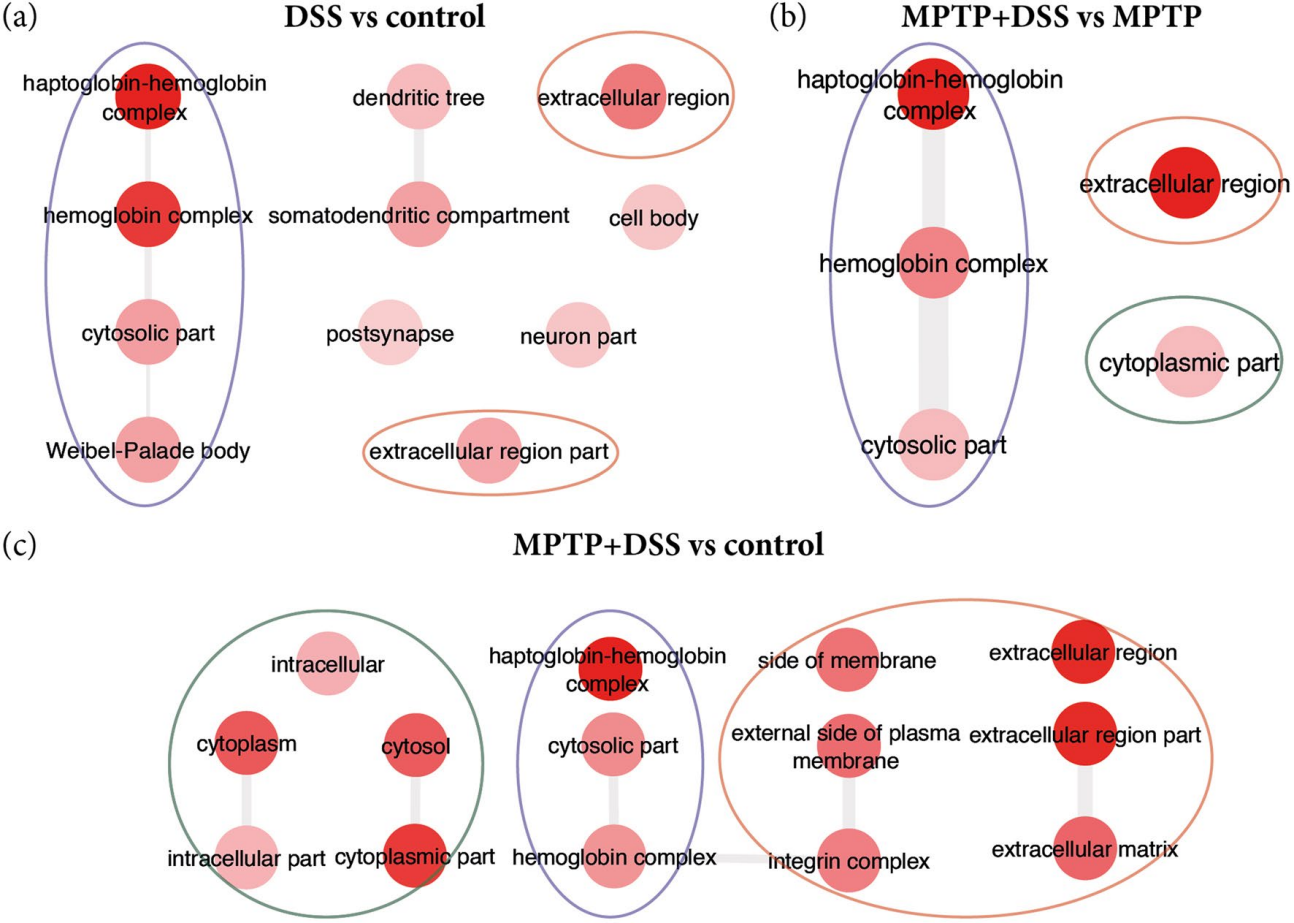
Summary of the main GO terms obtained for cellular component in the comparisons for DSS vs control (**a**), MPTP + DSS vs MPTP (**b**) and MPTP + DSS vs control (**c**). The color of the circles correspond to different groups of annotations: purple for haptoblobin-hemoglobin complex, orange for extracellular region and green for intracellular part.

Unlike in the category of biological process where in all groups came out significant up-regulation of the DEGs; in the cellular component were significantly observed only in the comparisons: DSS vs control, MPTP + DSS vs control and MPTP + DSS vs MPTP (Fig. 5). Interestingly, in the three comparisons (animals treated with DSS) the groups of genes related to haptoblobin-hemoglobin complex and the extracellular region stand out. In addition, when animals are treated together with MPTP (Fig. 5b and c.i, Table 4), changes are also observed in the intracellular part.

**Table 4:** The enriched GO terms for the DEGs of DSS and MPTP vs control, respectively.

GO Term	Description	Corrected *p*-value	DEGs count
**DSS vs control**			
GO:0031838	Haptoglobin–hemoglobin complex	7.02E−07	4
GO:0005833	Hemoglobin complex	1.46E−06	4
GO:0005576	Extracellular region	1.86E−04	20
GO:0044445	Cytosolic part	2.26E−03	6
GO:0036477	Somatodendritic compartment	2.77E−03	11
GO:0033093	Weibel-palade body	2.77E−03	2
GO:0005615	Extracellular space	2.77E−03	13
GO:0044421	Extracellular region part	3.42E−03	14
**MPTP + DSS vs MPTP**			
GO:0005576	Extracellular region	2.42E−06	39
GO:0031838	Haptoglobin–hemoglobin complex	2.42E−06	4
GO:0044421	Extracellular region part	3.43E−06	31
GO:0005615	Extracellular space	3.43E−06	28
GO:0005833	Hemoglobin complex	2.15E−03	3
**MPTP + DSS vs MPTP**			
GO:0044421	Extracellular region part	6.98E−07	104
GO:0005615	Extracellular space	6.98E−07	96
GO:0005576	Extracellular region	1.73E−06	122
GO:0044444	Cytoplasmic part	1.73E−06	302
GO:0005737	Cytoplasm	3.19E−05	360
GO:0005829	Cytosol	1.01E−04	147
GO:0031838	Haptoglobin–hemoglobin complex	3.64E−04	5
GO:0031012	Extracellular matrix	6.95E−04	30
GO:0009986	Cell surface	7.29E−04	53
GO:0008305	Integrin complex	2.41E−03	7
GO:0044445	Cytosolic part	3.07E−03	20
GO:0098636	Protein complex involved in cell adhesion	3.58E−03	7
GO:0005833	Hemoglobin complex	5.81E−03	4
GO:0009897	External side of plasma membrane	7.66E−03	30
GO:0098552	Side of membrane	7.66E−03	37

### Molecular function

**Figure 6:**
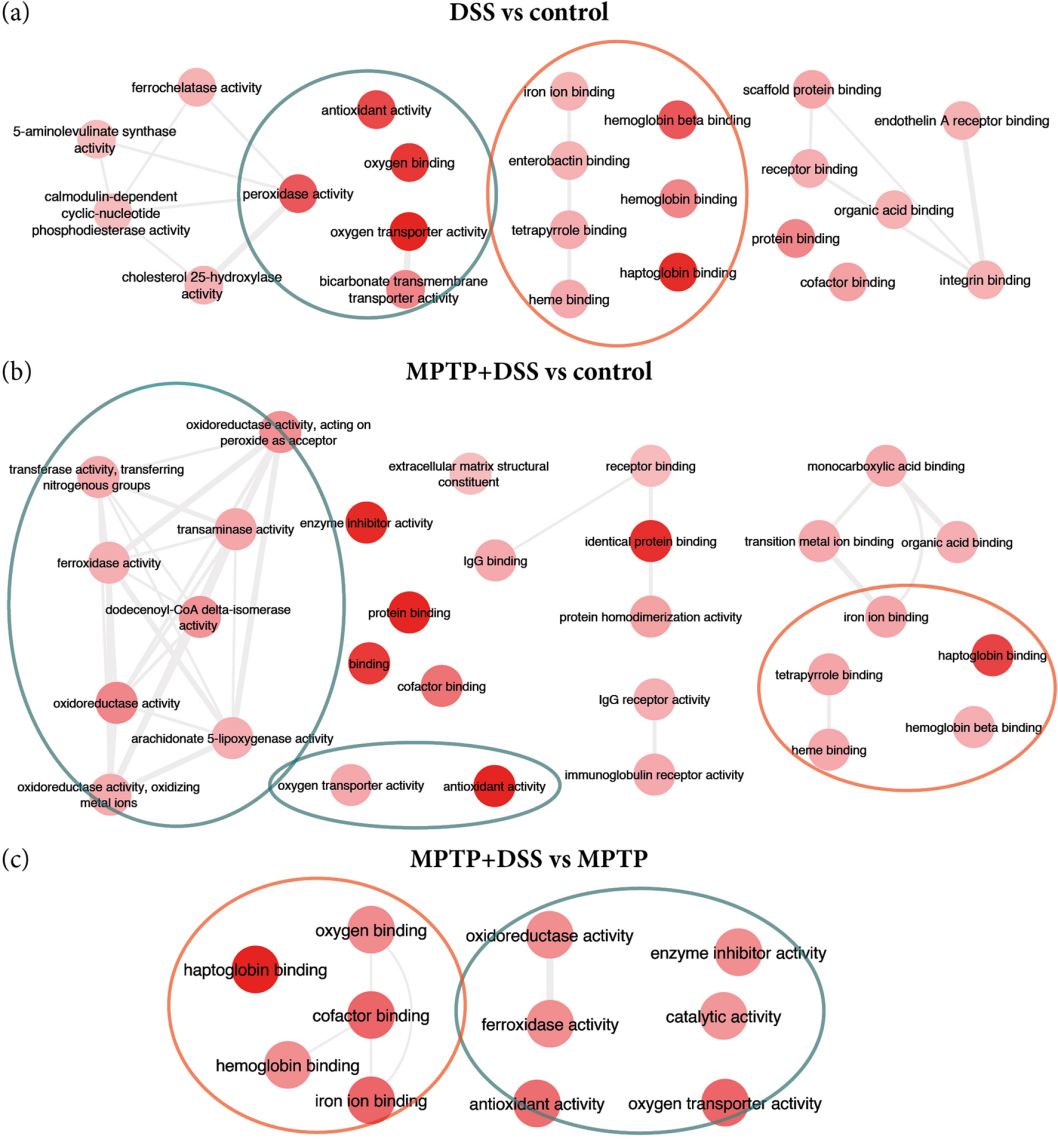
Summary of the main GO terms obtained for molecular function in the comparisons for DSS vs control (**a**), MPTP + DSS vs control (**b**) and MPTP + DSS vs MPTP (**c**).

Regarding molecular function GO annotations, we found terms from up-regulated DEGs mainly implicated in antioxidant activity and haptoglobin binding in the comparisons for DSS vs control, MPTP + DSS vs control and MPTP + DSS vs MPTP (Fig. 6, Table 5).

**Table 5:** The most significant enriched GO terms for the DEGs of DSS and MPTP vs control and MPTP + DSS vs MPTP.

GO term	Description	Corrected *p*-value	DEGs count
**DSS vs control**^a^			
GO:0031720	Haptoglobin binding	9.59E−06	4
GO:0005344	Oxygen carrier activity	3.37E−06	4
GO:0019825	Oxygen binding	2.73E−05	4
GO:0016209	Antioxidant activity	9.96E−05	5
GO:0140104	Molecular carrier activity	1.63E−04	4
GO:0004601	Peroxidase activity	2.19E−04	4
GO:0031722	Hemoglobin beta binding	2.19E−04	2
GO:0016684	Oxidoreductase activity, acting on peroxide as acceptor	2.52E−04	4
GO:0031721	Hemoglobin alpha binding	1.02E−03	2
GO:0030492	Hemoglobin binding	2.78E−03	2
GO:0015106	Bicarbonate transmembrane transporter activity	2.78E−03	2
GO:0005515	Protein binding	2.78E−03	35
GO:0005452	Inorganic anion exchanger activity	7.66E−03	2
**MPTP + DSS vs control**^a^			
GO:0042802	Identical protein binding	7.99E−07	97
GO:0016209	Antioxidant activity	8.02E−07	16
GO:0005515	Protein binding	2.33E−06	348
GO:0030414	Peptidase inhibitor activity	1.78E−05	22
GO:0004857	Enzyme inhibitor activity	1.93E−05	31
GO:0061134	Peptidase regulator activity	4.69E−05	23
GO:0004866	Endopeptidase inhibitor activity	6.02E−05	20
GO:0061135	Endopeptidase regulator activity	1.28E−04	20
GO:0048037	Cofactor binding	3.16E−04	37
GO:0005488	Binding	4.53E−04	460
GO:0016491	Oxidoreductase activity	5.62E−04	46
GO:0003824	Catalytic activity	1.12E−03	209
GO:0030234	Enzyme regulator activity	1.12E−03	50
GO:0042803	Protein homodimerization activity	1.22E−03	48
GO:0004869	Cysteine-type endopeptidase inhibitor activity	1.35e−03	10
GO:0016722	Oxidoreductase activity, oxidizing metal ions	1.50E−03	6
GO:0016684	Oxidoreductase activity, acting on peroxide as acceptor	1.75E−03	9
GO:0005506	Iron ion binding	1.75E−03	18
GO:0004322	Ferroxidase activity	2.04E−03	5
GO:0016724	Oxidoreductase activity, oxidizing metal ions, oxygen as acceptor	2.04E−03	5
GO:1901567	Fatty acid derivative binding	2.53E−03	7
GO:0031720	Haptoglobin binding	3.21E−03	4
GO:0046906	Tetrapyrrole binding	3.49E−03	16
GO:0004165	Dodecenoyl-coa delta-isomerase activity	3.73E−03	3
GO:0004601	Peroxidase activity	4.13E−03	8
GO:0020037	Heme binding	6.13E−03	15
GO:0043177	Organic acid binding	6.15E−03	17
GO:0046914	Transition metal ion binding	6.71E−03	50
**MPTP + DSS vs MPTP**^a^			
GO:0031720	Haptoglobin binding	4.89E−04	3

^a^Results shown with corrected *p*-value ≤ 1E−03.

### Reactome enriched analysis

The data obtained from the Reactome annotations divided the comparisons into different events in the same scenario (striatum level). Firstly, DSS compared to control stand out mechanisms related to heme dysfunction (as scavenging of heme from plasma or heme biosynthesis) possibly due to the bleeding produced by the DSS administration. Since free heme promotes the conversion of low density lipoproteins into cytotoxic products, make it toxic, it is also observed immune system processes up-regulation (Table 6).

**Table 6:** Functional enriched Reactome annotations for all the comparisons.

Reactome code	Description	Corrected *p*-value	DEGs count
**DSS vs control**			
1247673	Erythrocytes take up oxygen and release carbon dioxide	7.33E−08	5
1237044	Erythrocytes take up carbon dioxide and release oxygen	1.92E−07	5
1480926	O_2_/CO_2_ exchange in erythrocytes	1.92E−07	5
2168880	Scavenging of heme from plasma	8.25E−05	5
2173782	Binding and uptake of ligands by scavenger receptors	1.52E−04	5
189451	Heme biosynthesis	1.08E−02	2
189445	Metabolism of porphyrins	2.54E−02	2
216083	Integrin cell surface interactions	3.02E−02	3
168256	Immune system	3.02E−02	13
**MPTP vs control**			
140342	Apoptosis induced DNA fragmentation	1.45E−05	5
2559584	Formation of senescence-associated heterochromatin foci (SAHF)	4.68E−05	5
2559586	DNA damage/telomere stress induced senescence	4.58E−03	6
75153	Apoptotic execution phase	5.18E−03	5
**MPTP + DSS vs control**			
168256	Immune system	1.48E−07	111
168249	Innate immune system	1.14E−06	73
6798695	Neutrophil degranulation	2.75E−06	47
1247673	Erythrocytes take up oxygen and release carbon dioxide	2.91E−04	4
198933	Immunoregulatory interactions between a lymphoid and a Non-lymphoid cell	2.12E−03	19
5686938	Regulation of TLR by endogenous ligand	2.12E−03	6
804914	Transport of fatty acids	5.34E−03	4
425397	Transport of vitamins, nucleosides, and related molecules	8.93E−03	8
8978868	Fatty acid metabolism	1.33E−02	19
109582	Hemostasis	3.06E−02	39
216083	Integrin cell surface interactions	4.19E−02	10
1236975	Antigen processing-cross presentation	4.45E−02	7
2142688	Synthesis of 5-eicosatetraenoic acids	4.68E−02	3
**MPTP + DSS vs MPTP**			
174577	Activation of C3 and C5	7.20E−03	3
1247673	Erythrocytes take up oxygen and release carbon dioxide	1.21E−02	3
1480926	O_2_/CO_2_ exchange in erythrocytes	1.79E−02	3
1237044	Erythrocytes take up carbon dioxide and release oxygen	1.79E−02	3

Regarding MPTP compared to control, we observed two main pathways: (i) programmed cell death by apoptosis induced DNA fragmentation, and (ii) cellular responses to external stimuli that implicated DNA damage/telomerase stress induced senescence (Table 6).

On the other hand, the last events are exacerbated when comparing MPTP + DSS with both control and MPTP mice, which are the immune system processes. Specifically, it is observed: (i) the innate immune system response, with the up-regulation of the neutrophil degranulation and the toll-like receptor cascade (regulation of TLR by endogenous ligand) and, (ii) the adaptive immune system response, with the Class I MHC mediated antigen processing and presentation (antigen processing cross presentation) and immunoregulatory interactions between a lymphoid and a non-lymphoid cell (Table 6).

## Discussion

Prior work have remarked the involvement of systemic inflammation in the development of PD. The findings from the present work extend those published reports through a bioinformatics approach. We aimed to elucidate in depth the molecular and cellular mechanisms triggered after a systemic insult in the striatum of mice untreated and treated with MPTP. The importance of specifically studying the effect of both toxins in the striatum is based on previous data obtained by immunohistochemical techniques in which we observed exacerbation of the inflammatory events when combining both toxins^8^.

The results from this study provide novel information about the DEGs in the striatum of mice intoxicated only with DSS. It is clear to observe the up-regulation of processes related to detoxification and inflammatory mechanisms (Fig. 3, Table 3) mainly triggered by the bleeding and lesion of the mucosal surface caused by the administration of DSS^14^. This is the first study to our knowledge to describe the molecular and cellular mechanisms activated in the striatum after a local insult in the colon.

Otherwise, when comparing MPTP mice with the control group, we found biological processes related to the response to unfolded protein (Fig. 3b, Table 2). Interestingly, these results are in accordance with the observations of Fornai and colleagues who described that the administration of MPTP reduces the protein degradation function of the striatal ubiquitin–proteasome system^15^. Later on, it was added that this could lead the accumulation of unfolded proteins and consequently, activates stress-induced cell death mechanisms^16^. Moreover, it was demonstrated that oxidative stress processes may participate in the formation of cross-linked protein aggregates^17^. In these animals, we also noticed DEGs associated with an attempt to regulate negatively the apoptotic signalling pathway and positively the activation of the inflammatory response (Table 3). Interestingly, the data from Reactome analysis showed annotations related to the execution of apoptosis mechanisms induced by DNA fragmentation and the induction of cellular senescence. These results are in line with those reported to another study that suggests that the nature and severity of DNA damage may determine the cellular response^18^. More research is needed to clarify how is the interplay of both routes after a DNA insult^19^.

Most notably were the observations that resulted from the comparison between MPTP + DSS and control animals. As this work indicates, we obtained an exacerbation of the up-regulated DEGs in all the analysis related to detoxification, oxidative stress and immune system processes.

Our results not only reinforce those results reported in the literature but also extend detailed information on the contribution of systemic inflammation to the progression of the neurodegeneration associated with Parkinson’s disease. Although our aim was highly achieved, we are aware of some intrinsic limitations such as the number of animals used in this type of studies^20,21^ or the focus in one brain area. The relevance of this lies in the contribution of novel and detailed information that describes different expression profiles that can be used as a guide for further and specific analysis. We advise that future work should evaluate upregulated pathways in different brain areas over time. In this way, we will be able to identify promising therapeutic targets that prevent the contribution of inflammatory processes in the progression of Parkinson’s disease.

## Conclusions

Altogether, we provide functional and comprehensive bioinformatics analyses of the deleterious effect of the systemic inflammation in the striatum of MPTP intoxicated mice. Interestingly, the data showed in this study describes a scenario that becomes more complex when combining both treatments. Thus, the processes related to inflammation and oxidative stress are exacerbated resulting in a significant up-regulation of the cell death mechanisms.

## Supplementary information

Supplementary Information 1.

Supplementary Information 2.

## Data Availability

All data generated or analysed during this study are included in this published article [and its supplementary information files].
