# Supplementary material for: Identification of differentially expressed genes profiles in a combined mouse model of Parkinsonism and colitis
Source: Sci Rep. 2020 Aug 4;10:13147. doi: 10.1038/s41598-020-69695-4 (PMC7403295; doi:10.1038/s41598-020-69695-4)
Supplement: Supplementary file 1 — Supplementary Information 1. [file 41598_2020_69695_MOESM1_ESM.docx]

**SUPPLEMENTARY INFORMATION**

**Identification of differentially expressed genes profiles in a combined mouse model of Parkinsonism and colitis.**

Gil-Martinez, AL^1,2^; Cuenca-Bermejo L^1,2^; Gonzalez-Cuello AM^1,2^; Sanchez-Rodrigo C^1,2^; Parrado A^2^; Vyas S^3^; Fernandez-Villalba E^1,2,^*; Herrero MT^1,2,^*

^1^Clinical and Experimental Neuroscience Group (NiCE), Institute for Aging Research, School of Medicine, Campus Mare Nostrum, University of Murcia, 30100, Murcia, Spain.

^2^Biomedical Research Institute of Murcia (IMIB-Arrixaca), Campus of Health Sciences, University of Murcia, 30120, Murcia, Spain.

^3^Institute of Biology Paris Seine, Gene Regulation and Adaptive Behaviours team, Department of Neuroscience Paris Seine, Sorbonne Université, CNRS UMR 8246 & INSERM U1130, 9 Quai Saint Bernard, F-75005, Paris, France

*Corresponding authors: María Trinidad Herrero; [mtherrer@um.es](mailto:mtherrer@um.es); Emiliano Fernandez-Villalba, [emiliano.fernandez@carm.es](mailto:emiliano.fernandez@carm.es)

**Supplementary File 1: Identification of DEGs in the experimental groups (DSS, MPTP and MPTP+DSS) compared to Control.**


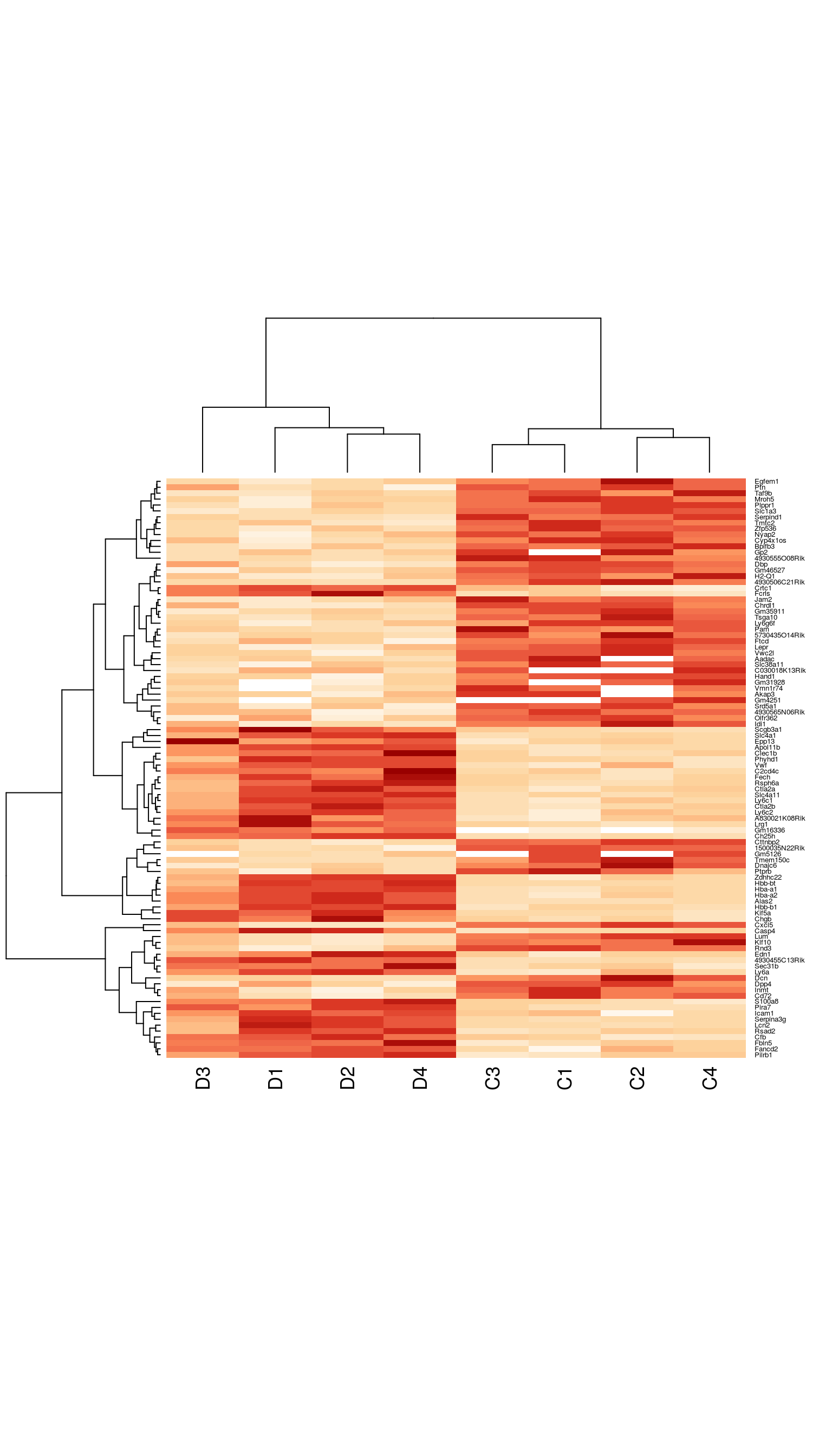


**Supplementary Figure 1.1.** The cluster heat map of differentially expressed genes (DEGs) of individual samples from DSS (D1-D4) and control (C1-C4) animals. Horizontal axis stands for samples of microarray data and vertical axis stands for DEGs. The upper half stands for down-regulated DEGs; data column of Parkinson’s disease (PD) samples was mainly green and healthy control (HC) samples was mainly red. The lower half stands for up- regulated DEGs; data column of PD samples was mainly red and HC samples was mainly green PPI.

| **Supplementary Table 1.1.**: Downregulated and upregulated DEGs in DSS compared to Control. | | | | | |
| --- | --- | --- | --- | --- | --- |
| **DOWNREGULATED DEGs** | | | **UPREGULATED DEGs** | | |
| **ProbeName** | **Gene Symbol** | **D_Ctrl** | **ProbeName** | **Gene Symbol** | **D_Ctrl** |
| A_66_P139224 | 4930555O08Rik | -3,758770295 | A_55_P1998591 | Fam43b | 0,250499947 |
| A_55_P2005705 | Gm4251 | -3,650740518 | A_66_P119187 | 4930519L02Rik | 0,277830849 |
| A_55_P2509028 | C030018K13Rik | -3,611919688 | A_52_P138806 | Dlgap3 | 0,310376954 |
| A_66_P115446 | Gm5126 | -3,269570276 | A_51_P353914 | Icam5 | 0,311952458 |
| A_51_P394115 | Aadac | -2,904275707 | A_55_P2052281 | Rnf208 | 0,315642761 |
| A_55_P2727439 | Gp2 | -2,478382264 | A_55_P1981929 | Scg5 | 0,331965737 |
| A_51_P370008 | Ceacam18 | -2,373602471 | A_51_P421303 | Caly | 0,341264902 |
| A_55_P2156268 | Bpifb3 | -2,231281178 | A_52_P49457 | Fbxl16 | 0,343670265 |
| A_55_P2549581 | Gm31928 | -2,227712985 | A_52_P49378 | Kif1a | 0,349306711 |
| A_51_P215475 | Ptprb | -2,156336594 | A_55_P1974827 | Pde1b | 0,349522271 |
| A_55_P2232297 | Vwc2l | -2,142591412 | A_51_P271425 | Lhfpl4 | 0,349809809 |
| A_51_P209413 | Akap3 | -2,12013798 | A_52_P465980 | Cplx1 | 0,353205121 |
| A_51_P332506 | Vmn1r74 | -2,044256168 | A_55_P2142439 | Echdc2 | 0,353482234 |
| A_52_P142827 | Slc38a11 | -2,013683741 | A_51_P181341 | Necab2 | 0,366352409 |
| A_66_P104552 | Slc1a3 | -1,841663918 | A_55_P2007630 | Sez6l2 | 0,371880043 |
| A_66_P108556 | Cyp4x1os | -1,820573074 | A_55_P2162910 | Rtn1 | 0,373099877 |
| A_51_P342549 | Hand1 | -1,739327595 | A_65_P01584 | Meg3 | 0,381403629 |
| A_52_P446457 | Ly6g6f | -1,534895599 | A_55_P2132512 | Ngef | 0,387633825 |
| A_55_P1990032 | Cxcl5 | -1,43820577 | A_65_P01437 | Pnmal2 | 0,39377938 |
| A_51_P106779 | Cttnbp2 | -1,426249807 | A_55_P2396375 | 1810073O08Rik | 0,412200645 |
| A_51_P167527 | Lum | -1,40148356 | A_52_P598309 | Zfas1 | 0,419747984 |
| A_55_P2094123 | Mroh5 | -1,330860402 | A_66_P136801 | Peg13 | 0,444775813 |
| A_66_P116936 | 4930565N06Rik | -1,298008651 | A_51_P220681 | Aldoc | 0,450301489 |
| A_55_P2157794 | Nyap2 | -1,133649244 | A_51_P111902 | Slc22a17 | 0,452800937 |
| A_51_P154379 | Tmem150c | -1,049940316 | A_51_P191669 | Chgb | 0,454056067 |
| A_51_P413740 | Ftcd | -1,039271952 | A_55_P2112170 | Crtc1 | 0,506991578 |
| A_51_P420415 | Srd5a1 | -0,936624458 | A_55_P2042146 | Fech | 0,517214235 |
| A_55_P2022074 | Klf10 | -0,93643227 | A_51_P107020 | Kif5a | 0,560560456 |
| A_55_P2741439 | Zfp536 | -0,906138133 | A_55_P2064771 | Ly6c1 | 0,576391256 |
| A_51_P444148 | Tmtc2 | -0,876811195 | A_52_P5579 | 4930455C13Rik | 0,584231207 |
| A_52_P25251 | Plppr1 | -0,863275434 | A_55_P2091461 | Casp4 | 0,597591384 |
| A_55_P1978502 | H2-Q1 | -0,858521732 | A_55_P2741920 | Fcrls | 0,649502428 |
| A_51_P389120 | Egfem1 | -0,856027057 | A_51_P265495 | Ly6a | 0,658374849 |
| A_55_P2256163 | 4930506C21Rik | -0,830299936 | A_51_P197528 | Ly6c2 | 0,684550155 |
| A_52_P573867 | Gm46527 | -0,826033131 | A_51_P104783 | Rsph6a | 0,71690266 |
| A_52_P592749 | Taf9b | -0,818284361 | A_66_P121976 | Slc4a11 | 0,742761329 |
| A_55_P2121729 | Gm35911 | -0,775769747 | A_55_P1978191 | C2cd4c | 0,762259867 |
| A_51_P334104 | Dcn | -0,756054946 | A_52_P288050 | Fbln5 | 0,791972206 |
| A_66_P105424 | Dnajc6 | -0,746219567 | A_51_P115005 | Edn1 | 0,801397462 |
| A_66_P107959 | 5730435O14Rik | -0,74374635 | A_55_P2026632 | Pilrb1 | 0,850158067 |
| A_55_P2177910 | Lepr | -0,723663328 | A_51_P327451 | Alas2 | 0,861106283 |
| A_55_P2733797 | Pam | -0,685187069 | A_66_P105958 | Sec31b | 0,879632672 |
| A_52_P537907 | Tsga10 | -0,629501691 | A_55_P2744479 | Phyhd1 | 0,880775968 |
| A_52_P674808 | Chrdl1 | -0,615171369 | A_51_P183239 | Fancd2 | 0,884135806 |
| A_55_P2726960 | 1500035N22Rik | -0,608291615 | A_55_P2718900 | Vwf | 0,896489681 |
| A_55_P2718945 | Dpp4 | -0,57752654 | A_51_P326191 | Serpina3g | 1,037165382 |
| A_66_P110662 | Ptn | -0,575732111 | A_66_P103894 | Zdhhc22 | 1,063114073 |
| A_55_P2086785 | Olfr362 | -0,568876871 | A_55_P1955656 | Ctla2a | 1,106654173 |
| A_51_P162162 | Inmt | -0,566087666 | A_51_P489522 | Ctla2b | 1,107329844 |
| A_51_P468140 | Serpind1 | -0,551501728 | A_55_P2108151 | Hbb-b1 | 1,13135067 |
| A_55_P2028961 | Idi1 | -0,515073024 | A_55_P2079579 | Pira7 | 1,2815342 |
| A_51_P180492 | Dbp | -0,509346367 | A_51_P233583 | Clec1b | 1,304725947 |
| A_51_P284946 | Rnd3 | -0,492119124 | A_55_P1973809 | Hbb-bt | 1,327238283 |
| A_51_P418375 | Jam2 | -0,490111742 | A_51_P413866 | Cfb | 1,382687152 |
| A_51_P292736 | Cd72 | -0,458535833 | A_51_P408506 | Icam1 | 1,481071901 |
| A_51_P216005 | Col4a5 | -0,410401527 | A_55_P1962303 | Hba-a1 | 1,589615617 |
| A_51_P312336 | Slc14a1 | -0,409086321 | A_55_P1962299 | Hba-a2 | 1,664567263 |
| A_55_P1961335 | Ctsk | -0,402754756 | A_55_P2238875 | A830021K08Rik | 1,863884427 |
| A_51_P485791 | Cyp51 | -0,400717072 | A_51_P110341 | Scgb3a1 | 1,970752899 |
| A_55_P2044385 | Fgfbp3 | -0,347506116 | A_51_P346938 | Lrg1 | 2,008363285 |
| A_51_P450365 | Txnrd3 | -0,336950659 | A_51_P510156 | Lcn2 | 2,009118707 |
| A_66_P108468 | 2610528A11Rik | -0,327884002 | A_52_P275069 | Epp13 | 2,061523154 |
| A_55_P2084631 | Hist1h2an | -0,252562646 | A_52_P670026 | Rsad2 | 2,158543459 |
|  |  |  | A_51_P126236 | Gm14461 | 2,220263142 |
|  |  |  | A_51_P112966 | Ch25h | 2,909180886 |
|  |  |  | A_55_P2510282 | Gm16336 | 3,002582226 |
|  |  |  | A_51_P196972 | Slc4a1 | 3,26108001 |
|  |  |  | A_51_P256827 | S100a8 | 3,737741807 |
|  |  |  | A_55_P2822972 | Apol11b | 5,665521207 |


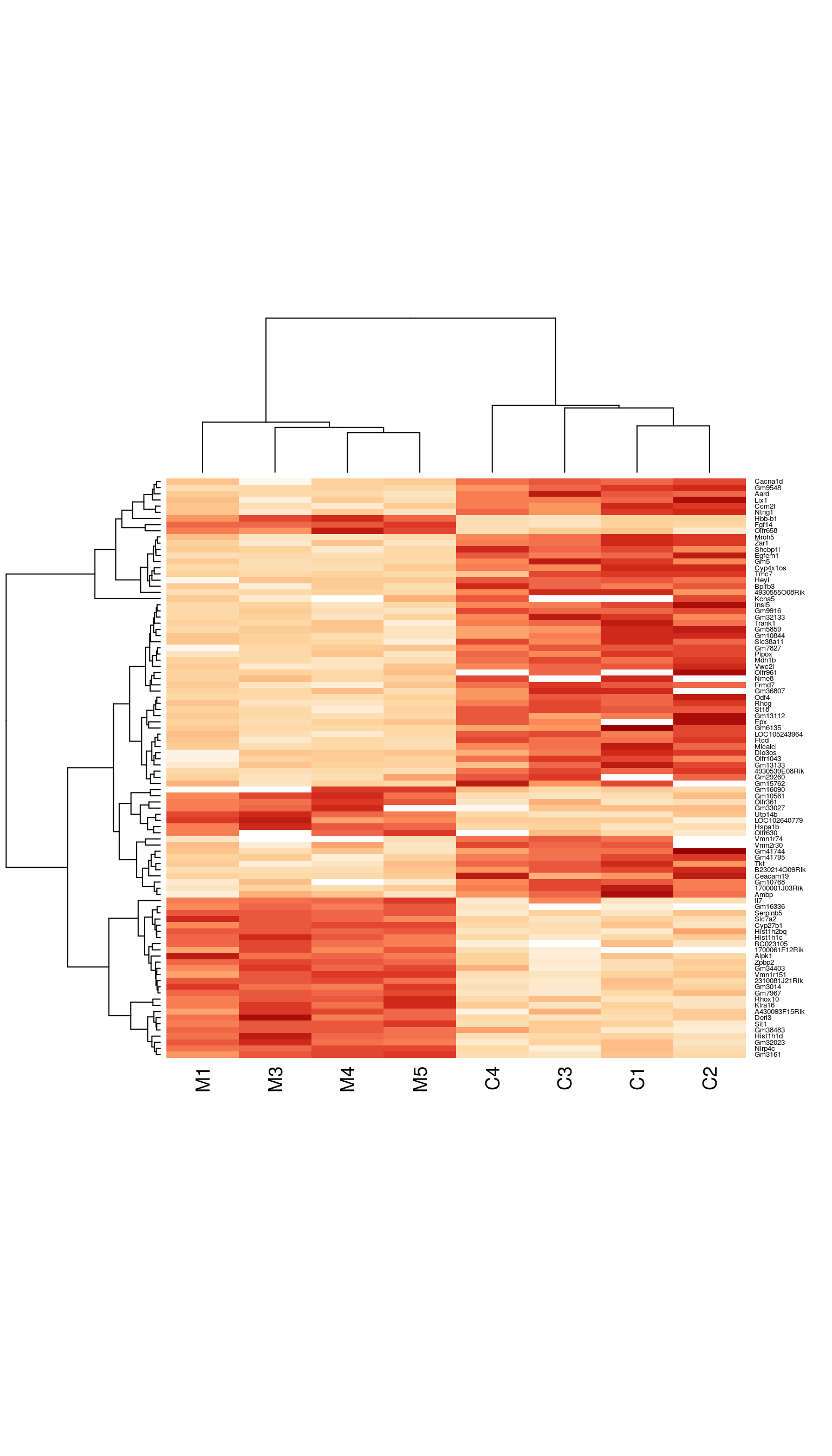


**Supplementary Figure 1.2.** The cluster heat map of differentially expressed genes (DEGs) of individual samples from MPTP (M1-M4) and control (C1-C4) animals. Horizontal axis stands for samples of microarray data and vertical axis stands for DEGs. The upper half stands for down-regulated DEGs; data column of Parkinson’s disease (PD) samples was mainly green and healthy control (HC) samples was mainly red. The lower half stands for up- regulated DEGs; data column of PD samples was mainly red and HC samples was mainly green PPI.

| **Supplementary Table 1.2.**: Downregulated and upregulated DEGs in MPTP compared to Control. | | | | | |
| --- | --- | --- | --- | --- | --- |
| **DOWNREGULATED DEGs** | | | **UPREGULATED DEGs** | | |
| **ProbeName** | **Gene Symbol** | **M_Ctrl** | **ProbeName** | **Gene Symbol** | **M_Ctrl** |
| A_55_P2097853 | Gm13769 | -4,574082996 | A_52_P67200 | Stt3b | 0,203262757 |
| A_51_P332506 | Vmn1r74 | -3,666628068 | A_51_P118415 | Scpep1 | 0,224433188 |
| A_66_P139224 | 4930555O08Rik | -3,568522385 | A_55_P2043267 | Cdc42se1 | 0,240211966 |
| A_51_P387097 | Mbd3l1 | -3,440801096 | A_51_P136888 | Rb1 | 0,243076179 |
| A_52_P58283 | Kcna5 | -3,376082845 | A_51_P502608 | Wwtr1 | 0,256624925 |
| A_51_P230055 | Olfr961 | -2,826508927 | A_55_P2185273 | Umps | 0,263800771 |
| A_51_P370008 | Ceacam18 | -2,823958711 | A_55_P2720386 | March7 | 0,269318925 |
| A_55_P1996957 | Vmn2r30 | -2,73314259 | A_51_P239766 | Plcd1 | 0,277846786 |
| A_51_P421479 | Zdhhc11 | -2,634035364 | A_55_P1976689 | Snrnp35 | 0,279479012 |
| A_55_P2638733 | Frmd7 | -2,606328317 | A_55_P2170414 | Zfp180 | 0,281788296 |
| A_51_P386625 | Epx | -2,591912979 | A_52_P35581 | Nqo2 | 0,28442798 |
| A_66_P109491 | Gm36807 | -2,469398234 | A_52_P233335 | Pphln1 | 0,284521506 |
| A_52_P295225 | Nme8 | -2,336554234 | A_52_P152283 | Gabpa | 0,285260302 |
| A_55_P2156268 | Bpifb3 | -2,284640075 | A_52_P589268 | Smg7 | 0,294070248 |
| A_66_P119917 | Gm6135 | -2,192633502 | A_51_P345896 | Pafah2 | 0,29864312 |
| A_66_P114270 | Gm29260 | -2,176282412 | A_52_P91757 | Bcorl1 | 0,306091934 |
| A_55_P2071360 | Gm15762 | -2,105699212 | A_51_P128987 | Akr1b8 | 0,308420627 |
| A_51_P179480 | 4930539E08Rik | -1,955087437 | A_51_P187697 | Mgme1 | 0,314023125 |
| A_51_P467238 | Ceacam19 | -1,941838284 | A_66_P119400 | Slco2b1 | 0,31572573 |
| A_52_P142827 | Slc38a11 | -1,909466255 | A_51_P105078 | S100a4 | 0,317078901 |
| A_52_P337259 | Heyl | -1,827937492 | A_55_P2021378 | Wapl | 0,322456954 |
| A_66_P108556 | Cyp4x1os | -1,792307565 | A_66_P106589 | Exd2 | 0,33046635 |
| A_55_P2232297 | Vwc2l | -1,760731219 | A_51_P442759 | Arhgef19 | 0,330698315 |
| A_55_P2198946 | Gm10768 | -1,749842769 | A_55_P2216561 | 2500002B13Rik | 0,331771689 |
| A_52_P522372 | Aard | -1,55462464 | A_55_P1992045 | Msi2 | 0,332151855 |
| A_55_P2834604 | Gm13133 | -1,520852473 | A_55_P1984406 | Lrrk2 | 0,335004775 |
| A_66_P139662 | Gm5 | -1,516230503 | A_52_P614517 | Rhobtb1 | 0,336830655 |
| A_55_P2296320 | Gm13112 | -1,487398082 | A_52_P307142 | 2210408F21Rik | 0,338670618 |
| A_55_P1981836 | Ccm2l | -1,457679268 | A_55_P2002351 | Lats2 | 0,341043254 |
| A_55_P2846580 | St18 | -1,418582135 | A_66_P111562 | Ccnd1 | 0,342908297 |
| A_55_P2083454 | Gm10844 | -1,357715395 | A_55_P2032960 | Brap | 0,3477092 |
| A_52_P213889 | Tmc7 | -1,301975672 | A_51_P520936 | Bcar3 | 0,349164287 |
| A_51_P133911 | Olfr1043 | -1,289260601 | A_55_P1960167 | Bcat2 | 0,349462954 |
| A_55_P2083153 | 1700001J03Rik | -1,24354091 | A_52_P326657 | Fam167b | 0,351571153 |
| A_55_P2251121 | B230214O09Rik | -1,237241811 | A_55_P2902672 | Myadm | 0,351968037 |
| A_51_P410772 | Rhcg | -1,222901411 | A_51_P496804 | Adat2 | 0,355896573 |
| A_51_P266077 | Shcbp1l | -1,2096011 | A_51_P415688 | Gng12 | 0,357230993 |
| A_55_P2934222 | Gm5859 | -1,201545004 | A_51_P479464 | Cdk19 | 0,365810532 |
| A_66_P136423 | Gm41744 | -1,201167049 | A_51_P319453 | Zfp143 | 0,366362351 |
| A_51_P513000 | Ntng1 | -1,185374775 | A_51_P293688 | Rab32 | 0,368553298 |
| A_51_P365139 | Micalcl | -1,174225445 | A_52_P319073 | Lrrc57 | 0,371597463 |
| A_55_P2094123 | Mroh5 | -1,167847831 | A_51_P106538 | Htra3 | 0,373310602 |
| A_51_P413740 | Ftcd | -1,149737849 | A_55_P2803608 | Cpa4 | 0,374425242 |
| A_51_P389120 | Egfem1 | -1,148064711 | A_55_P2043657 | Zfp677 | 0,37447693 |
| A_66_P102172 | Dio3os | -1,142806387 | A_55_P2067438 | Def8 | 0,377803535 |
| A_66_P124223 | Gm9548 | -1,139186151 | A_52_P357611 | Neu3 | 0,378859428 |
| A_51_P145785 | Ambp | -1,065250155 | A_55_P2785472 | Ormdl2 | 0,383183193 |
| A_51_P145380 | Zar1 | -1,062924876 | A_55_P1999364 | Krt85 | 0,383573896 |
| A_51_P227077 | Mdh1b | -1,042963137 | A_52_P511269 | Mlec | 0,383786541 |
| A_55_P2255325 | Insl5 | -0,978521658 | A_51_P248786 | Ccdc80 | 0,387594996 |
| A_55_P1977239 | Gm9916 | -0,92766748 | A_51_P139745 | Acat3 | 0,390013063 |
| A_55_P2125376 | Cacna1d | -0,913821336 | A_52_P40954 | Dubr | 0,392071253 |
| A_66_P104807 | LOC105243964 | -0,897565109 | A_55_P2002757 | Blnk | 0,394522924 |
| A_51_P394515 | Tkt | -0,874966664 | A_51_P418168 | Manf | 0,394942026 |
| A_51_P351062 | Odf4 | -0,873564127 | A_51_P479321 | Acss1 | 0,398086965 |
| A_55_P1991039 | Gm7827 | -0,862066428 | A_55_P2004263 | BC025920 | 0,401187664 |
| A_55_P2550442 | Gm32133 | -0,862032737 | A_55_P2053491 | Pdia6 | 0,409390031 |
| A_51_P298266 | Lix1 | -0,852993083 | A_51_P347764 | Noc3l | 0,410330168 |
| A_51_P337195 | Pipox | -0,849559148 | A_51_P437240 | Emp2 | 0,411618364 |
| A_55_P2154782 | Gm41795 | -0,845513652 | A_52_P580707 | Zfp781 | 0,414446592 |
| A_51_P508115 | Trank1 | -0,841982481 | A_55_P2371281 | 9030601B04Rik | 0,41486394 |
| A_51_P219594 | 1700123K08Rik | -0,838170878 | A_55_P2061391 | Rhobtb3 | 0,417971079 |
| A_66_P132695 | 1700015F17Rik | -0,810891531 | A_51_P346668 | Irf5 | 0,423137218 |
| A_55_P2055970 | LOC102639013 | -0,79424329 | A_55_P2720430 | Nfe2l2 | 0,429119042 |
| A_55_P2152607 | Cyp4a12b | -0,790456905 | A_51_P394802 | Fam111a | 0,433021226 |
| A_66_P108216 | Klhl40 | -0,771228432 | A_55_P1987974 | Glipr1l3 | 0,438475866 |
| A_55_P2944462 | Ccdc116 | -0,763232701 | A_51_P137150 | Dhx8 | 0,438631543 |
| A_66_P125352 | B230303O12Rik | -0,753507374 | A_55_P2178800 | Ugt1a10 | 0,440029467 |
| A_55_P2177910 | Lepr | -0,72706471 | A_55_P1973563 | 5730559C18Rik | 0,442934104 |
| A_66_P107959 | 5730435O14Rik | -0,725555056 | A_66_P131467 | Mfap4 | 0,443009172 |
| A_52_P592749 | Taf9b | -0,71990322 | A_55_P2734317 | Vcl | 0,447009594 |
| A_55_P1987146 | Adgrf4 | -0,715180819 | A_51_P160754 | Apobec1 | 0,45106303 |
| A_66_P120992 | Rnf152 | -0,714890915 | A_55_P2931347 | Hsph1 | 0,453898886 |
| A_55_P2582888 | Gm30609 | -0,708471461 | A_51_P463628 | Vis1 | 0,456215054 |
| A_55_P2499253 | Rsg1 | -0,701416285 | A_55_P2502806 | Dthd1 | 0,456267116 |
| A_66_P105424 | Dnajc6 | -0,687358319 | A_51_P228295 | Mpzl1 | 0,464705439 |
| A_52_P338266 | Rec114 | -0,683004883 | A_55_P2796612 | Bag3 | 0,467363873 |
| A_55_P2116993 | Hnrnpdl | -0,67737371 | A_55_P2880322 | Gm12522 | 0,46888252 |
| A_66_P112004 | LOC108167931 | -0,672001733 | A_52_P198435 | Rasgrp3 | 0,469045506 |
| A_51_P231597 | Nkain3 | -0,653935544 | A_55_P2803234 | Mtap | 0,475563826 |
| A_55_P2033795 | Fabp6 | -0,651940627 | A_55_P1973906 | Trp53inp1 | 0,478266616 |
| A_55_P2728628 | Sall3 | -0,649973707 | A_55_P2115875 | 1700012D14Rik | 0,479331238 |
| A_52_P228899 | Scn3b | -0,624299483 | A_66_P127452 | Hk3 | 0,482176719 |
| A_66_P104483 | Ciart | -0,617069217 | A_51_P431737 | Cth | 0,482765414 |
| A_66_P120446 | Kcnb1 | -0,599702108 | A_51_P430929 | Fam20a | 0,483142413 |
| A_55_P2484565 | Pxt1 | -0,59856563 | A_55_P2728573 | Adamts7 | 0,483846366 |
| A_55_P1976764 | Polr3e | -0,595415271 | A_55_P2718333 | Rcc1 | 0,486728566 |
| A_52_P102432 | Dsg1c | -0,591508768 | A_55_P1994032 | Xbp1 | 0,487422326 |
| A_51_P497768 | Gpat2 | -0,590107448 | A_55_P2222437 | D430040D24Rik | 0,487787293 |
| A_55_P2713390 | Sema3c | -0,582281049 | A_51_P167263 | Cd5 | 0,491071361 |
| A_52_P520037 | Rimbp2 | -0,580770667 | A_52_P106620 | Tnfrsf11b | 0,495704765 |
| A_51_P170827 | Asb5 | -0,576074333 | A_52_P502577 | S1pr3 | 0,499279996 |
| A_66_P109457 | 2410021H03Rik | -0,573357042 | A_55_P1978424 | Bcl2a1d | 0,505261002 |
| A_52_P419455 | Adora2b | -0,562796036 | A_55_P2744772 | Treml2 | 0,508050958 |
| A_51_P499698 | Asprv1 | -0,562793539 | A_55_P2340101 | LOC102643247 | 0,510552953 |
| A_66_P107014 | Fam135b | -0,559320006 | A_51_P177984 | Fam234a | 0,510769394 |
| A_55_P2106434 | Zfp114 | -0,558451933 | A_51_P288876 | Tmem45a | 0,511828011 |
| A_51_P335569 | Slco1a4 | -0,558126164 | A_55_P2102540 | Olfml3 | 0,514220138 |
| A_55_P2107367 | Pbx3 | -0,552163253 | A_55_P2039284 | Hspb1 | 0,518920088 |
| A_51_P265465 | Abhd3 | -0,54027453 | A_51_P263591 | Pank1 | 0,522728196 |
| A_52_P679711 | Tmem269 | -0,500658737 | A_55_P2232057 | AU022793 | 0,524545894 |
| A_55_P1966977 | Sntb1 | -0,499850876 | A_66_P132518 | Osbpl1a | 0,537310855 |
| A_55_P2072233 | Zcchc12 | -0,4975688 | A_52_P23674 | Adgrf3 | 0,538791613 |
| A_52_P110052 | Ackr1 | -0,484946359 | A_55_P2032718 | Klra9 | 0,545246813 |
| A_66_P138459 | Gm11738 | -0,483684672 | A_51_P115178 | Scara3 | 0,547310812 |
| A_51_P450365 | Txnrd3 | -0,479391782 | A_66_P116886 | Gm20627 | 0,548007745 |
| A_52_P657360 | Tnni1 | -0,469935541 | A_52_P162695 | Pvr | 0,55139123 |
| A_55_P2729115 | Sema4b | -0,468502572 | A_51_P122246 | Creld2 | 0,551694005 |
| A_52_P706060 | Mex3a | -0,467206224 | A_52_P434841 | Coa4 | 0,553405667 |
| A_51_P461429 | Cyp7b1 | -0,456983133 | A_55_P2724789 | Arhgap5 | 0,555108504 |
| A_51_P209327 | Apln | -0,456240302 | A_52_P438036 | Lpar4 | 0,558021765 |
| A_51_P340829 | AA986860 | -0,450394721 | A_66_P110906 | 3110083C13Rik | 0,558726424 |
| A_55_P2647945 | Plekha1 | -0,44679849 | A_55_P2083764 | Rbm12 | 0,561716578 |
| A_51_P362202 | Mob3c | -0,444853049 | A_55_P2892369 | Hmgb2 | 0,564522064 |
| A_55_P2058726 | Hap1 | -0,444771311 | A_51_P451574 | Acot1 | 0,574152169 |
| A_52_P609024 | Cenpw | -0,433706746 | A_55_P2732788 | Rmi2 | 0,575028229 |
| A_66_P136102 | Lefty2 | -0,430786812 | A_55_P2049567 | Pld2 | 0,587425816 |
| A_55_P2230506 | 4931402H11Rik | -0,429589092 | A_66_P132249 | Akr1c13 | 0,593829698 |
| A_51_P412914 | Efs | -0,408458992 | A_52_P439502 | Plekhh1 | 0,603977786 |
| A_51_P488991 | Oaf | -0,407901559 | A_55_P1961034 | Gm13272 | 0,606092154 |
| A_55_P2721008 | Strada | -0,399077074 | A_55_P1999883 | Fthl17-ps1 | 0,609655843 |
| A_51_P397934 | Grin3b | -0,391239064 | A_52_P484838 | Rfxank | 0,609767189 |
| A_52_P883941 | Gm46304 | -0,386654652 | A_51_P497882 | Creb3l4 | 0,613178262 |
| A_52_P646755 | Tex30 | -0,383744463 | A_55_P2787236 | Gm32885 | 0,613666243 |
| A_66_P118579 | 3300002A11Rik | -0,377051098 | A_55_P2600332 | Lactb2 | 0,614431883 |
| A_55_P2747220 | Sephs2 | -0,375859947 | A_55_P2743421 | Slc35d2 | 0,618564305 |
| A_55_P2148624 | Gpr61 | -0,373844012 | A_66_P117032 | C4b | 0,621964422 |
| A_55_P2727654 | Gbe1 | -0,372952949 | A_55_P1978191 | C2cd4c | 0,62277928 |
| A_55_P2066707 | Trim27 | -0,369357718 | A_66_P114333 | Tlr12 | 0,623002694 |
| A_66_P137881 | Gm39121 | -0,360063525 | A_66_P115451 | Gm5122 | 0,631018115 |
| A_52_P685821 | Tmem245 | -0,356848669 | A_55_P2744012 | Exd1 | 0,639717445 |
| A_55_P2060193 | Mcf2l | -0,354572528 | A_66_P116502 | Il17rc | 0,656572927 |
| A_55_P2129856 | Mtfmt | -0,354223464 | A_66_P105132 | Oas1g | 0,664325646 |
| A_55_P2154228 | Ncapd3 | -0,353348713 | A_51_P280446 | Sdf2l1 | 0,670414639 |
| A_55_P2419483 | 4732460I02Rik | -0,346069197 | A_66_P136719 | Gm12532 | 0,675961366 |
| A_55_P2275402 | 9330177L23Rik | -0,337275547 | A_51_P147274 | Clec4a3 | 0,678541172 |
| A_51_P495269 | Lor | -0,322576003 | A_55_P2044932 | Gpr84 | 0,679668283 |
| A_51_P205286 | Pcsk4 | -0,318368362 | A_52_P550932 | H1f0 | 0,692002204 |
| A_51_P378545 | Rbpms2 | -0,312165533 | A_51_P497171 | Ly9 | 0,692095152 |
| A_51_P301435 | B3galt5 | -0,31107041 | A_51_P341203 | Cyp3a41a | 0,692812611 |
| A_51_P251357 | Ctps | -0,298680463 | A_55_P2069221 | Prr11 | 0,694626809 |
| A_55_P2769911 | Nxt1 | -0,287216516 | A_55_P2076772 | Hspa5 | 0,704905359 |
| A_55_P1985351 | Slc35f2 | -0,269353103 | A_51_P208240 | Tnfsf14 | 0,716182107 |
| A_66_P137121 | Cks1b | -0,264637179 | A_51_P276235 | Pnpla7 | 0,722139248 |
| A_55_P2106922 | Arfgap3 | -0,260263594 | A_51_P517695 | Ly6f | 0,74892345 |
| A_51_P405565 | Msh6 | -0,258782519 | A_51_P464822 | Hist1h1e | 0,764480896 |
| A_51_P279127 | Fam173a | -0,237954382 | A_51_P513682 | Nuf2 | 0,766020675 |
| A_55_P2339650 | Hnrnpl | -0,237194278 | A_51_P183239 | Fancd2 | 0,778118355 |
| A_52_P195772 | Rbmx | -0,233819717 | A_51_P357735 | Inhbe | 0,783833651 |
| A_51_P137388 | Zadh2 | -0,2181971 | A_55_P2023021 | Gm6961 | 0,797146801 |
|  |  |  | A_55_P2028288 | Nhlrc4 | 0,807417791 |
|  |  |  | A_55_P2783103 | LOC735298 | 0,8075859 |
|  |  |  | A_66_P121526 | Kank4os | 0,809591193 |
|  |  |  | A_55_P2109128 | Hist1h2be | 0,814992397 |
|  |  |  | A_51_P275496 | Gm39749 | 0,821868012 |
|  |  |  | A_55_P2026632 | Pilrb1 | 0,83115692 |
|  |  |  | A_52_P40504 | LOC102640779 | 0,854645054 |
|  |  |  | A_55_P2480694 | Hist1h2bq | 0,862057794 |
|  |  |  | A_55_P2108151 | Hbb-b1 | 0,86686962 |
|  |  |  | A_55_P2028734 | Klra16 | 0,879698414 |
|  |  |  | A_55_P2743349 | Derl3 | 0,882838586 |
|  |  |  | A_55_P2016882 | Nlrp4c | 0,906965442 |
|  |  |  | A_55_P1954436 | Gm7967 | 0,909526596 |
|  |  |  | A_51_P144632 | Sit1 | 0,939812094 |
|  |  |  | A_55_P2849189 | Slc7a2 | 0,94569224 |
|  |  |  | A_55_P2184567 | Gm3161 | 0,965415653 |
|  |  |  | A_66_P100197 | Gm34403 | 0,979506374 |
|  |  |  | A_55_P2088237 | Cyp27b1 | 0,981190007 |
|  |  |  | A_55_P1964896 | Gm3014 | 1,009977193 |
|  |  |  | A_66_P125111 | Gm38483 | 1,030132385 |
|  |  |  | A_52_P262930 | 2310081J21Rik | 1,03982854 |
|  |  |  | A_55_P1988985 | Vmn1r151 | 1,07436856 |
|  |  |  | A_51_P516133 | Hist1h1c | 1,098635592 |
|  |  |  | A_66_P110116 | Gm10561 | 1,110103029 |
|  |  |  | A_52_P526740 | A430093F15Rik | 1,179159033 |
|  |  |  | A_55_P1996893 | Zpbp2 | 1,209391207 |
|  |  |  | A_66_P118021 | Hspa1b | 1,244869927 |
|  |  |  | A_55_P2505350 | Utp14b | 1,275175602 |
|  |  |  | A_55_P2829197 | Gm32023 | 1,27768012 |
|  |  |  | A_51_P126236 | Gm14461 | 1,469666427 |
|  |  |  | A_66_P138201 | Rhox10 | 1,657244967 |
|  |  |  | A_51_P187602 | Serpinb5 | 1,718739317 |
|  |  |  | A_55_P1985164 | Olfr658 | 1,754047919 |
|  |  |  | A_55_P2730641 | Alpk1 | 1,924748736 |
|  |  |  | A_55_P2510282 | Gm16336 | 1,957637612 |
|  |  |  | A_52_P10683 | Hist1h1d | 1,960458708 |
|  |  |  | A_55_P2098408 | Olfr630 | 1,969392492 |
|  |  |  | A_51_P364871 | Olfr361 | 2,234800289 |
|  |  |  | A_51_P434737 | BC023105 | 2,279281037 |
|  |  |  | A_66_P121674 | 1700061F12Rik | 2,389754665 |
|  |  |  | A_66_P130204 | Gm16090 | 2,540562804 |
|  |  |  | A_55_P2547227 | Gm33027 | 2,728433965 |
|  |  |  | A_52_P168549 | Fgf14 | 2,901601765 |
|  |  |  | A_55_P2732016 | Il7 | 3,11629314 |
|  |  |  | A_66_P108587 | 7330404K18Rik | 4,64480375 |


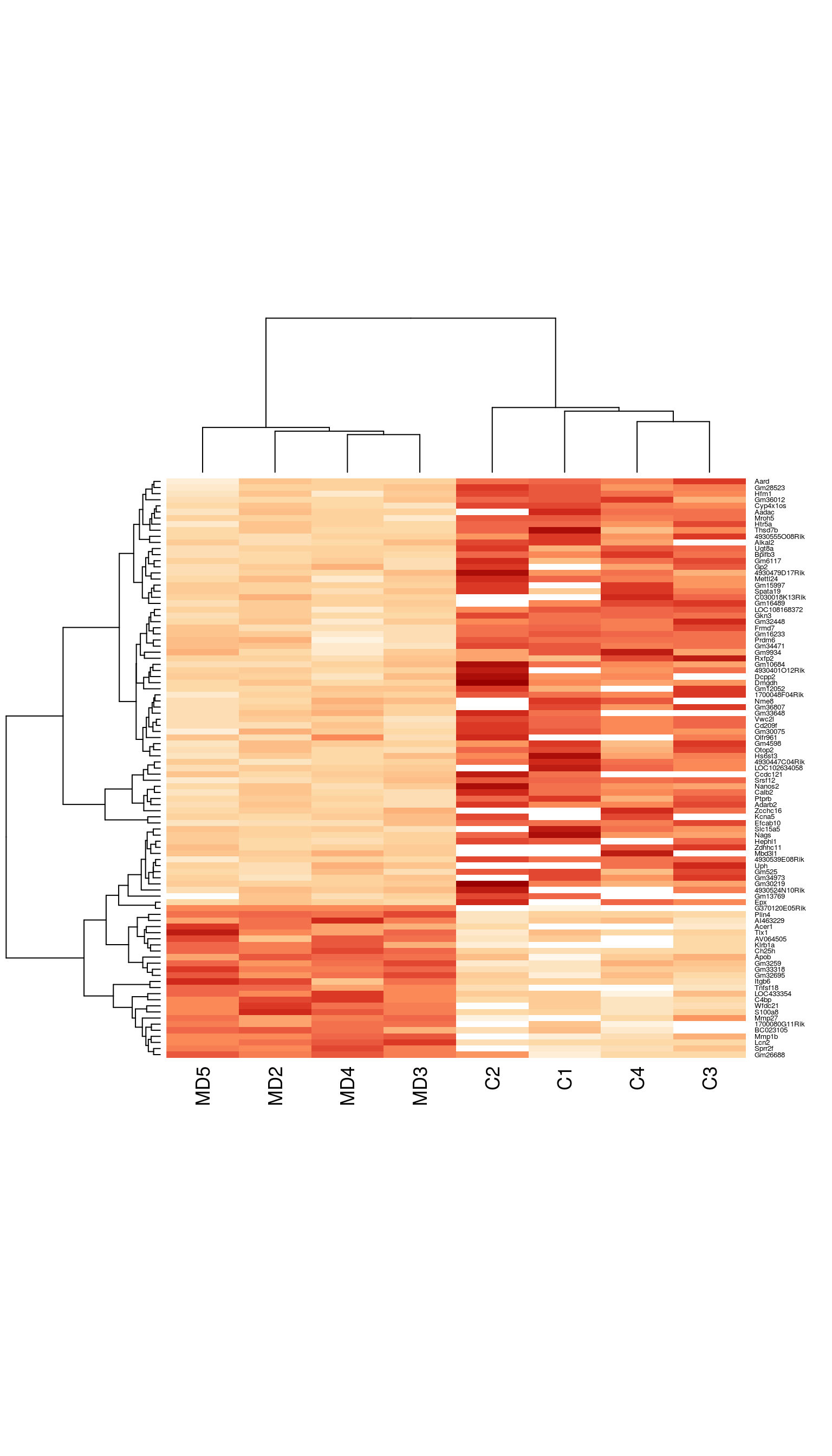


**Supplementary Figure 1.3.** The cluster heat map of differentially expressed genes (DEGs) of individual samples from MPTP+DSS (MD1-MD4) and control (C1-C4) animals. Horizontal axis stands for samples of microarray data and vertical axis stands for DEGs. The upper half stands for down-regulated DEGs; data column of Parkinson’s disease (PD) samples was mainly green and healthy control (HC) samples was mainly red. The lower half stands for up- regulated DEGs; data column of PD samples was mainly red and HC samples was mainly green PPI.

| **Supplementary Table 1.3.**: Downregulated and upregulated DEGs in MPTP+DSS compared to Control. | | | | | |
| --- | --- | --- | --- | --- | --- |
| **DEGs DOWNREGULATED** | | | **DEGs UPREGULATED** | | |
| **ProbeName** | **Gene Symbol** | **MD_Ctrl** | **ProbeName** | **Gene Symbol** | **MD_Ctrl** |
| A_51_P230347 | Srsf12 | -3,966849751 | A_55_P2110081 | Cisd3 | 0,17958396 |
| A_55_P2097853 | Gm13769 | -3,837502024 | A_55_P2830645 | Wipf2 | 0,18460554 |
| A_66_P139224 | 4930555O08Rik | -3,827533158 | A_55_P2822526 | Hs1bp3 | 0,18547298 |
| A_66_P101881 | Gm15997 | -3,767125462 | A_51_P441263 | Rpl37a | 0,18688244 |
| A_51_P433908 | 4930524N10Rik | -3,698435496 | A_55_P2168549 | Ap5b1 | 0,19282003 |
| A_55_P2005705 | Gm4251 | -3,598551598 | A_51_P446229 | Rpl18a | 0,1933983 |
| A_66_P137676 | Hephl1 | -3,527239386 | A_55_P2052281 | Rnf208 | 0,20064776 |
| A_66_P118098 | Gm12052 | -3,42145531 | A_55_P2043267 | Cdc42se1 | 0,20315153 |
| A_51_P402974 | Gm16489 | -3,287325754 | A_51_P119429 | Nckap1l | 0,20441196 |
| A_55_P2192617 | AI854905 | -3,222148143 | A_55_P2113066 | Zkscan5 | 0,20613736 |
| A_55_P2568181 | Gm33648 | -3,167715959 | A_52_P227307 | Mllt1 | 0,20846778 |
| A_55_P2786960 | Dcpp2 | -3,121653013 | A_65_P00792 | COX1 | 0,2127326 |
| A_51_P387097 | Mbd3l1 | -3,118989534 | A_55_P2163622 | Rps27 | 0,21283128 |
| A_52_P1148292 | Gm20275 | -3,108886943 | A_55_P2038299 | Rpl31 | 0,21556196 |
| A_55_P1998781 | Zcchc16 | -3,013626408 | A_66_P114386 | Rnpep | 0,21558751 |
| A_51_P386625 | Epx | -3,009931436 | A_51_P461779 | Ppp2r2c | 0,21592335 |
| A_66_P116152 | Gm16233 | -2,931853493 | A_55_P2134387 | Olfr544 | 0,21662602 |
| A_55_P2509028 | C030018K13Rik | -2,907453705 | A_55_P2721169 | Ccndbp1 | 0,21669761 |
| A_66_P137954 | Gm34471 | -2,863894536 | A_52_P368945 | Skap2 | 0,21708487 |
| A_52_P139438 | Alkal2 | -2,818403456 | A_55_P2625808 | Ppp1r8 | 0,21791636 |
| A_51_P273679 | Dmgdh | -2,7693935 | A_55_P2079020 | Snhg7os | 0,22248635 |
| A_66_P109491 | Gm36807 | -2,716111943 | A_66_P139576 | Ralbp1 | 0,22657518 |
| A_55_P2145511 | Gm36012 | -2,633801684 | A_52_P657593 | Fxr1 | 0,22861472 |
| A_55_P2797478 | Spata19 | -2,592737016 | A_55_P2722170 | Snap47 | 0,22995446 |
| A_55_P2638733 | Frmd7 | -2,540253497 | A_55_P2846276 | Eif4b | 0,2314806 |
| A_55_P2087345 | Htr5a | -2,523043412 | A_55_P2067142 | Fau | 0,23221966 |
| A_55_P2077483 | Adarb2 | -2,520032949 | A_66_P129356 | 2810468N07Rik | 0,23343593 |
| A_52_P523399 | 4930447C04Rik | -2,506175637 | A_66_P134025 | Rps14 | 0,24016408 |
| A_51_P215475 | Ptprb | -2,479436714 | A_51_P442759 | Arhgef19 | 0,24041342 |
| A_51_P421479 | Zdhhc11 | -2,423048339 | A_66_P119400 | Slco2b1 | 0,2414309 |
| A_55_P2302978 | 1700048F04Rik | -2,411731404 | A_55_P2132512 | Ngef | 0,24152816 |
| A_55_P2509223 | 4930401O12Rik | -2,404850024 | A_66_P138287 | Gm15245 | 0,24169607 |
| A_51_P467629 | Efcab10 | -2,404395879 | A_51_P470311 | Cby1 | 0,24175052 |
| A_51_P230055 | Olfr961 | -2,39423084 | A_55_P2081790 | Slc44a1 | 0,24189399 |
| A_52_P58283 | Kcna5 | -2,382729324 | A_66_P136899 | Nadk | 0,24210944 |
| A_55_P2086860 | Ccdc121 | -2,30427938 | A_55_P2494518 | Arpc1a | 0,24305265 |
| A_55_P2556138 | Gm32448 | -2,303082751 | A_55_P2170414 | Zfp180 | 0,2434245 |
| A_55_P2094123 | Mroh5 | -2,294840786 | A_55_P2122938 | Wdyhv1 | 0,24352373 |
| A_52_P295225 | Nme8 | -2,290967425 | A_52_P604195 | Mbp | 0,2474577 |
| A_55_P2734767 | LOC102634058 | -2,28056295 | A_52_P268687 | Hipk1 | 0,24876919 |
| A_55_P2727439 | Gp2 | -2,245655563 | A_55_P2049549 | Rab22a | 0,24879254 |
| A_66_P110646 | Gm6117 | -2,240202552 | A_51_P279163 | Plcg2 | 0,25058213 |
| A_52_P302661 | Slc15a5 | -2,233885578 | A_66_P124084 | Eif3f | 0,25183003 |
| A_66_P119219 | 4930479D17Rik | -2,2288262 | A_55_P1953301 | Sord | 0,25366774 |
| A_55_P2731971 | Thsd7b | -2,221479311 | A_66_P116275 | Gm2629 | 0,25532602 |
| A_51_P506822 | Ugt8a | -2,198861833 | A_55_P1976689 | Snrnp35 | 0,25534742 |
| A_55_P2584627 | LOC108168372 | -2,189898549 | A_51_P386058 | Ppp2r5b | 0,25865509 |
| A_55_P2730348 | Gm10684 | -2,171683602 | A_66_P103269 | 2810001A02Rik | 0,25865974 |
| A_66_P112109 | Gm4598 | -2,166172125 | A_55_P2507365 | Wnt11 | 0,25961073 |
| A_55_P2118794 | Otop2 | -2,142507074 | A_51_P309158 | Snx20 | 0,25962912 |
| A_51_P179480 | 4930539E08Rik | -2,123467699 | A_51_P416137 | Slc31a2 | 0,26156853 |
| A_55_P2232297 | Vwc2l | -2,116698211 | A_52_P582309 | Nub1 | 0,26201149 |
| A_52_P384084 | Rxfp2 | -2,114423722 | A_51_P348397 | Hexim1 | 0,26320049 |
| A_55_P1965897 | Hs6st3 | -2,056295998 | A_51_P219662 | Pxdc1 | 0,26342809 |
| A_51_P311436 | Calb2 | -2,055441659 | A_55_P2903199 | Serpinb9 | 0,26640368 |
| A_66_P106959 | Gm34973 | -2,043140362 | A_55_P1976224 | Ckb | 0,26701212 |
| A_66_P119268 | Gm28523 | -2,024770411 | A_52_P686091 | Phf2 | 0,26811919 |
| A_55_P1984961 | Uph | -2,023862735 | A_66_P122756 | Gm12758 | 0,26815899 |
| A_52_P399095 | Mettl24 | -2,023220134 | A_51_P111902 | Slc22a17 | 0,26835683 |
| A_55_P2000543 | Cd209f | -2,000310059 | A_66_P127371 | Arntl | 0,26911105 |
| A_66_P108556 | Cyp4x1os | -1,998929509 | A_51_P487278 | Yipf3 | 0,26970707 |
| A_51_P267700 | Gkn3 | -1,994838293 | A_55_P2717598 | Wdsub1 | 0,27049391 |
| A_55_P2744543 | Hfm1 | -1,980485134 | A_51_P396917 | Zfyve21 | 0,27575493 |
| A_66_P134326 | Gm30075 | -1,978195383 | A_55_P2129309 | Foxo3 | 0,27656081 |
| A_55_P2156268 | Bpifb3 | -1,971815997 | A_52_P303904 | Card6 | 0,27662181 |
| A_55_P2017977 | Gm525 | -1,95675758 | A_52_P67964 | Ss18 | 0,27711579 |
| A_52_P522372 | Aard | -1,953400601 | A_55_P1984406 | Lrrk2 | 0,28042879 |
| A_52_P559076 | Prdm6 | -1,947246687 | A_66_P125196 | Notum | 0,28250618 |
| A_52_P487436 | Nags | -1,940672946 | A_55_P2036567 | Vav1 | 0,28261414 |
| A_55_P1989195 | Gm30219 | -1,900990595 | A_52_P185343 | Gna13 | 0,28403407 |
| A_51_P394115 | Aadac | -1,898046344 | A_55_P2846586 | Nop53 | 0,28466266 |
| A_66_P131137 | Gm9934 | -1,89641312 | A_55_P2045216 | 4921531P14Rik | 0,28604111 |
| A_55_P2096071 | Nanos2 | -1,874591129 | A_55_P2917815 | Hmgcl | 0,28637672 |
| A_55_P2077153 | Slc27a2 | -1,872656034 | A_51_P427674 | Cpt1a | 0,28694021 |
| A_66_P140913 | Itga8 | -1,845838455 | A_55_P2909620 | Rpl7 | 0,2870448 |
| A_51_P216456 | Tac1 | -1,844890649 | A_51_P368823 | Grb7 | 0,28720978 |
| A_55_P1981836 | Ccm2l | -1,833783797 | A_55_P2096599 | Tsc22d4 | 0,28736764 |
| A_52_P142827 | Slc38a11 | -1,827959092 | A_66_P128150 | Trpt1 | 0,28744887 |
| A_51_P249608 | Trdn | -1,818864348 | A_66_P112553 | Ppp1r7 | 0,28778411 |
| A_51_P476654 | Pcdha11 | -1,816206423 | A_66_P128101 | Pdlim1 | 0,28802089 |
| A_52_P52849 | Cpxm2 | -1,804724262 | A_51_P302056 | Tubd1 | 0,2889291 |
| A_55_P2346619 | 8030498J20Rik | -1,799838473 | A_52_P657435 | Psmc5 | 0,28984696 |
| A_51_P472955 | Btbd16 | -1,79701803 | A_55_P2077628 | Dcxr | 0,29006888 |
| A_55_P2156006 | Kcnk15 | -1,782491733 | A_51_P358354 | Jam3 | 0,29059538 |
| A_55_P2930341 | Grm4 | -1,764296819 | A_55_P2076524 | 1700028I16Rik | 0,29138428 |
| A_55_P2126192 | Lgr5 | -1,759939258 | A_52_P163626 | Cfhr2 | 0,29322259 |
| A_55_P2008860 | Gm15520 | -1,70738161 | A_55_P2047514 | Ypel3 | 0,29339344 |
| A_66_P134394 | Pde6c | -1,705606565 | A_55_P2027594 | Taf3 | 0,29364047 |
| A_51_P209413 | Akap3 | -1,692640074 | A_55_P1979645 | S100a1 | 0,29458968 |
| A_52_P213550 | Chrna5 | -1,686590326 | A_52_P237102 | Dpy19l3 | 0,29683247 |
| A_55_P2575930 | Gm32359 | -1,666880015 | A_66_P133478 | Nsun5 | 0,29927099 |
| A_55_P2099665 | Gpr37 | -1,665366531 | A_51_P106047 | Tnfrsf1a | 0,29928467 |
| A_51_P472003 | Ttc22 | -1,664975875 | A_51_P381811 | 1700040A12Rik | 0,29989831 |
| A_55_P2094916 | Ocm | -1,661354754 | A_52_P128964 | Nhej1 | 0,30050155 |
| A_55_P2020731 | Brs3 | -1,643021837 | A_52_P475080 | Arsk | 0,30175912 |
| A_55_P2047111 | Gm30810 | -1,624594786 | A_51_P432511 | Afg1l | 0,30188933 |
| A_55_P2016114 | Fasl | -1,62204154 | A_51_P394802 | Fam111a | 0,30235509 |
| A_55_P2305010 | A730089K16Rik | -1,619983709 | A_55_P2132932 | Mageb1 | 0,30266653 |
| A_55_P1990032 | Cxcl5 | -1,611222006 | A_55_P2719394 | Col6a3 | 0,30269752 |
| A_51_P370008 | Ceacam18 | -1,608089624 | A_51_P432380 | Aplp1 | 0,30273467 |
| A_66_P104552 | Slc1a3 | -1,602176733 | A_65_P17773 | Imp4 | 0,30429749 |
| A_55_P2296320 | Gm13112 | -1,596231553 | A_55_P2112085 | Vwa8 | 0,30479688 |
| A_55_P2054097 | Gm43263 | -1,578907055 | A_55_P2732738 | Gm16283 | 0,30634788 |
| A_55_P2723230 | Hspa12a | -1,575646713 | A_51_P124254 | Col4a1 | 0,30939868 |
| A_51_P513000 | Ntng1 | -1,563168278 | A_51_P482239 | Bmf | 0,30982963 |
| A_55_P1971834 | Muc15 | -1,557238278 | A_66_P127144 | Fuca1 | 0,31001978 |
| A_51_P389120 | Egfem1 | -1,546069309 | A_66_P134821 | 5330411J11Rik | 0,31021617 |
| A_51_P265806 | Clca3a2 | -1,542623131 | A_51_P120738 | P2ry14 | 0,31063693 |
| A_51_P332506 | Vmn1r74 | -1,542330766 | A_55_P2840380 | Il16 | 0,31160397 |
| A_55_P2157794 | Nyap2 | -1,527846723 | A_51_P421876 | Irf7 | 0,31187144 |
| A_66_P113459 | Tspan2os | -1,522815423 | A_55_P2491848 | Eci2 | 0,31212141 |
| A_55_P1963046 | Prl3c1 | -1,513757335 | A_51_P128987 | Akr1b8 | 0,31234557 |
| A_55_P2083454 | Gm10844 | -1,475159788 | A_52_P362757 | Col6a2 | 0,31281285 |
| A_55_P2168271 | 6430553K19Rik | -1,462672429 | A_51_P355360 | Jak3 | 0,31616291 |
| A_55_P2049226 | Allc | -1,460224509 | A_51_P145415 | Lpcat3 | 0,31621019 |
| A_55_P2199382 | Gm10635 | -1,459215513 | A_52_P426768 | Cited4 | 0,31621224 |
| A_52_P376374 | Kcnh7 | -1,456773707 | A_55_P2742136 | Matn2 | 0,31775822 |
| A_55_P2808675 | Pcdh20 | -1,447237287 | A_51_P445662 | Hsd17b4 | 0,31783944 |
| A_55_P2549581 | Gm31928 | -1,442407985 | A_51_P232399 | Acy3 | 0,31916518 |
| A_55_P2834604 | Gm13133 | -1,424755459 | A_55_P2480051 | Gm20594 | 0,31929884 |
| A_55_P2251121 | B230214O09Rik | -1,420808485 | A_55_P2043582 | 4930480E11Rik | 0,31946358 |
| A_66_P114887 | Gm38520 | -1,412319603 | A_55_P2112967 | Diaph1 | 0,31976708 |
| A_52_P213889 | Tmc7 | -1,408767967 | A_51_P146753 | Csf2rb2 | 0,32012734 |
| A_55_P2721718 | Kcnq3 | -1,39644206 | A_55_P2743401 | Krt16 | 0,32087954 |
| A_51_P183051 | Upb1 | -1,389315035 | A_51_P160754 | Apobec1 | 0,32175885 |
| A_52_P440621 | Rassf10 | -1,377720026 | A_66_P132232 | Ercc1 | 0,32208852 |
| A_51_P286423 | Foxd3 | -1,364949908 | A_55_P2902743 | Azin1 | 0,32255748 |
| A_52_P337259 | Heyl | -1,360956818 | A_51_P181341 | Necab2 | 0,32285968 |
| A_51_P444148 | Tmtc2 | -1,354784275 | A_51_P481482 | Dram1 | 0,32368319 |
| A_66_P101490 | Prox1 | -1,341408106 | A_51_P517843 | Glipr2 | 0,32397465 |
| A_55_P2279777 | 2810404M03Rik | -1,3297229 | A_66_P116390 | Cst3 | 0,32442588 |
| A_55_P2804020 | 9530080O11Rik | -1,328373203 | A_55_P2714812 | Wdr62 | 0,32452351 |
| A_55_P2724194 | Ccna1 | -1,322185852 | A_51_P105589 | Eci1 | 0,32501731 |
| A_51_P316310 | Btn1a1 | -1,319913326 | A_51_P296764 | Fcgr1 | 0,32900997 |
| A_52_P115950 | Gm35558 | -1,31987035 | A_55_P1953241 | Mafk | 0,32981082 |
| A_66_P133356 | Gm26843 | -1,316123303 | A_55_P2102135 | Rdh12 | 0,3300936 |
| A_55_P1996598 | Tmco5 | -1,312923694 | A_55_P2007630 | Sez6l2 | 0,33024093 |
| A_55_P2811769 | Mccc1os | -1,303529224 | A_55_P2895406 | Eml3 | 0,33072403 |
| A_51_P410772 | Rhcg | -1,302523769 | A_51_P219789 | H2-Q2 | 0,33230013 |
| A_66_P111911 | BB131254 | -1,302170158 | A_52_P552550 | Agtrap | 0,33278274 |
| A_55_P1978502 | H2-Q1 | -1,301079465 | A_55_P2740937 | Tnf | 0,33316521 |
| A_52_P248013 | Chsy3 | -1,30102763 | A_51_P242201 | Naaa | 0,33321781 |
| A_66_P124403 | Gm14342 | -1,291782121 | A_51_P187082 | G6pdx | 0,33542897 |
| A_51_P137336 | Cdh1 | -1,282215457 | A_66_P106702 | Cln6 | 0,33586291 |
| A_52_P487362 | Ppp4r4 | -1,276575705 | A_55_P2757871 | Emx2 | 0,33620606 |
| A_52_P599478 | Fam181a | -1,274579932 | A_55_P2739639 | Trpm4 | 0,33727176 |
| A_51_P145380 | Zar1 | -1,268342163 | A_55_P2025038 | Cpe | 0,33759393 |
| A_55_P2170817 | AI847159 | -1,268200771 | A_55_P2028415 | Hacd2 | 0,33769215 |
| A_66_P139662 | Gm5 | -1,267257128 | A_52_P8059 | Sphk2 | 0,33848949 |
| A_55_P2107045 | Myl4 | -1,264711114 | A_51_P405476 | Fcer1g | 0,33911395 |
| A_55_P1977239 | Gm9916 | -1,246370039 | A_55_P2011802 | Pdk2 | 0,33919458 |
| A_51_P342549 | Hand1 | -1,241417454 | A_55_P2126578 | Gm41515 | 0,33929075 |
| A_51_P442142 | Trp53tg5 | -1,241214021 | A_55_P2051313 | Gstk1 | 0,33931359 |
| A_52_P380301 | Unc5c | -1,240038659 | A_52_P117325 | Rbl2 | 0,34003078 |
| A_51_P358037 | Abi3bp | -1,220376814 | A_66_P119584 | Scnn1a | 0,34224524 |
| A_55_P2128591 | Klk6 | -1,218856478 | A_66_P127452 | Hk3 | 0,34510288 |
| A_51_P220317 | Cdca7l | -1,217965518 | A_55_P2023562 | Cd300c | 0,346447 |
| A_66_P130251 | Gm31763 | -1,216089921 | A_51_P291224 | Mobp | 0,34755364 |
| A_51_P298266 | Lix1 | -1,203531821 | A_52_P434841 | Coa4 | 0,34767119 |
| A_51_P101065 | Htr7 | -1,203219417 | A_51_P213030 | Macrod1 | 0,34789046 |
| A_55_P2008443 | Mybpc1 | -1,202613501 | A_51_P233478 | Usf1 | 0,34932492 |
| A_66_P131939 | Drd5 | -1,200075346 | A_55_P2929462 | Slco4a1 | 0,34947266 |
| A_52_P478394 | Dleu7 | -1,191715995 | A_51_P270436 | Ficd | 0,34947823 |
| A_55_P2074406 | Ldoc1 | -1,189307816 | A_55_P2083939 | Irf3 | 0,34955965 |
| A_52_P883714 | Epha5 | -1,188385672 | A_52_P326657 | Fam167b | 0,34981278 |
| A_55_P2713410 | Lingo2 | -1,176653933 | A_52_P138926 | Slc32a1 | 0,34983561 |
| A_51_P133911 | Olfr1043 | -1,173209058 | A_55_P2724974 | H2-T23 | 0,34984133 |
| A_55_P2256163 | 4930506C21Rik | -1,162833444 | A_66_P129722 | Eci3 | 0,35039858 |
| A_55_P2437763 | 8430431K14Rik | -1,159461272 | A_51_P141634 | Pskh1 | 0,35062848 |
| A_51_P376912 | Zfp783 | -1,14628904 | A_52_P612803 | Ccng1 | 0,35079887 |
| A_55_P2156712 | Gpr1 | -1,139232083 | A_55_P2021378 | Wapl | 0,35149586 |
| A_55_P2176185 | Pcdh15 | -1,121332665 | A_65_P17358 | C1qtnf1 | 0,35150206 |
| A_52_P817975 | Klf12 | -1,121142478 | A_52_P198885 | Slc14a2 | 0,35215906 |
| A_52_P248604 | Cdh5 | -1,111953607 | A_51_P494675 | Cotl1 | 0,35243112 |
| A_66_P136423 | Gm41744 | -1,110631547 | A_55_P2060101 | Tprgl | 0,35269722 |
| A_51_P167527 | Lum | -1,108063628 | A_52_P656024 | Sirt2 | 0,35315332 |
| A_55_P2744836 | Art3 | -1,101052779 | A_52_P106620 | Tnfrsf11b | 0,35354741 |
| A_55_P2730906 | Nalcn | -1,095404636 | A_55_P2080151 | Hspa2 | 0,35632764 |
| A_55_P2846580 | St18 | -1,089777326 | A_55_P2736430 | Cdk6 | 0,35642027 |
| A_51_P499082 | Igsf1 | -1,076327849 | A_51_P323712 | Agt | 0,35808584 |
| A_51_P266077 | Shcbp1l | -1,075496707 | A_55_P2042698 | Hsd17b10 | 0,35970344 |
| A_55_P2048194 | Wscd1 | -1,074439237 | A_55_P2088425 | Tcn2 | 0,360379 |
| A_55_P2054628 | Nrxn1 | -1,074142368 | A_51_P271503 | Il1r1 | 0,36053121 |
| A_51_P365604 | 9030612E09Rik | -1,066423474 | A_51_P337856 | Ubl7 | 0,36219262 |
| A_55_P2483194 | Wfdc18 | -1,064857967 | A_55_P2129354 | Pilra | 0,36446687 |
| A_55_P2130039 | 5830477G23Rik | -1,063235534 | A_66_P102823 | Trim71 | 0,36466348 |
| A_66_P132078 | Gm13016 | -1,063220956 | A_66_P126445 | Dand5 | 0,36570068 |
| A_55_P1966377 | 4930502E18Rik | -1,053434684 | A_52_P634522 | Mis18bp1 | 0,36656206 |
| A_66_P105318 | Gm13383 | -1,048527986 | A_52_P398989 | Cytip | 0,36665363 |
| A_52_P956261 | Ndnf | -1,046759368 | A_52_P263673 | Etfbkmt | 0,3672736 |
| A_52_P14569 | 1700101I11Rik | -1,044713827 | A_51_P415855 | Il6ra | 0,36779389 |
| A_55_P2742475 | Dock3 | -1,044251038 | A_55_P2427900 | Unc13c | 0,36847746 |
| A_66_P124223 | Gm9548 | -1,039279152 | A_51_P136508 | Avp | 0,36939982 |
| A_51_P154379 | Tmem150c | -1,038072713 | A_51_P410918 | Tor4a | 0,3696027 |
| A_51_P268617 | Cntnap4 | -1,033335319 | A_51_P174723 | Cd86 | 0,37012556 |
| A_51_P413740 | Ftcd | -1,024398784 | A_55_P2324425 | Adam12 | 0,37115676 |
| A_66_P102172 | Dio3os | -1,013172401 | A_55_P2501511 | Slc9a5 | 0,37149709 |
| A_55_P2719204 | Wscd2 | -1,012306819 | A_55_P2133983 | Gm436 | 0,37237972 |
| A_55_P2083153 | 1700001J03Rik | -1,010970451 | A_55_P2085333 | Fbxo44 | 0,37256002 |
| A_66_P116507 | Tmprss7 | -1,00548334 | A_51_P349546 | Cd109 | 0,37270689 |
| A_66_P111457 | Hecw1 | -1,002458211 | A_52_P59206 | Cst6 | 0,37353374 |
| A_52_P25251 | Plppr1 | -1,000577105 | A_52_P284889 | Prkcz | 0,37389006 |
| A_51_P383399 | Aldh1a7 | -0,994012929 | A_52_P639689 | Gm4907 | 0,37824969 |
| A_52_P405708 | Dcc | -0,987349058 | A_55_P2027822 | Sgf29 | 0,37906878 |
| A_52_P550124 | 1700112J16Rik | -0,983477076 | A_55_P2014987 | Gatsl2 | 0,37924083 |
| A_55_P2033215 | Cabp5 | -0,982625007 | A_51_P418116 | Tmem119 | 0,37930261 |
| A_51_P461894 | Tnnc1 | -0,972199749 | A_52_P281659 | Klf13 | 0,38145264 |
| A_55_P2069765 | Kiss1 | -0,968632736 | A_55_P2714035 | Krt83 | 0,38238415 |
| A_66_P128904 | Rnf165 | -0,965439995 | A_55_P2080163 | Carhsp1 | 0,38326375 |
| A_55_P2729863 | A730087J23Rik | -0,958154969 | A_51_P232207 | Hoxb6 | 0,38512875 |
| A_55_P2734277 | Ace2 | -0,946513817 | A_51_P233954 | Sesn1 | 0,38519389 |
| A_66_P118385 | Gm34567 | -0,944868276 | A_51_P103780 | Sft2d2 | 0,38554995 |
| A_55_P1963184 | Pgap1 | -0,943568476 | A_51_P260169 | Gstm5 | 0,38563651 |
| A_51_P268863 | Car10 | -0,938340585 | A_52_P641849 | Khnyn | 0,38724408 |
| A_52_P154101 | Calca | -0,937412841 | A_51_P451588 | Plekhb1 | 0,38865731 |
| A_55_P2034300 | Tmem40 | -0,937272163 | A_55_P1960167 | Bcat2 | 0,38887233 |
| A_55_P2255325 | Insl5 | -0,929154455 | A_51_P158584 | Poll | 0,38896108 |
| A_51_P362089 | Lrrc3b | -0,922114512 | A_51_P107020 | Kif5a | 0,39073963 |
| A_55_P1987709 | Ptprd | -0,92038895 | A_51_P148670 | Nudt7 | 0,39102571 |
| A_52_P130787 | Kcna6 | -0,911334074 | A_55_P2142439 | Echdc2 | 0,3911493 |
| A_66_P105424 | Dnajc6 | -0,90827678 | A_51_P125260 | Acaa2 | 0,39125998 |
| A_52_P592749 | Taf9b | -0,888376663 | A_55_P2013043 | Serpinb6b | 0,39195491 |
| A_66_P107959 | 5730435O14Rik | -0,88799694 | A_51_P513568 | Stx11 | 0,3937068 |
| A_51_P115626 | Shank3 | -0,885725583 | A_55_P2106058 | Gm11110 | 0,39445896 |
| A_55_P1961152 | Pou3f1 | -0,885300046 | A_55_P2055557 | Sdsl | 0,39481185 |
| A_55_P2935136 | LOC102640344 | -0,884117216 | A_52_P658122 | Ets2 | 0,39503847 |
| A_51_P106779 | Cttnbp2 | -0,884095216 | A_52_P123693 | E130304I02Rik | 0,39652268 |
| A_66_P121924 | A330043C09Rik | -0,874309216 | A_55_P1977802 | Izumo1r | 0,39709776 |
| A_55_P2608493 | Tro | -0,873028662 | A_55_P1973563 | 5730559C18Rik | 0,39722054 |
| A_52_P415155 | Wnt6 | -0,872747105 | A_66_P105033 | Gm29675 | 0,39753956 |
| A_51_P227662 | Dnah7a | -0,871171283 | A_55_P2053398 | Pirb | 0,39765529 |
| A_51_P160389 | 4930470F04Rik | -0,86917452 | A_52_P491569 | 1700017B05Rik | 0,40075096 |
| A_66_P124688 | BC049352 | -0,867956036 | A_66_P134479 | Glul | 0,4014323 |
| A_51_P401850 | Vmn1r225 | -0,86787716 | A_55_P2097828 | Gmfg | 0,40421321 |
| A_55_P2154782 | Gm41795 | -0,867297205 | A_52_P357133 | Selenom | 0,40493377 |
| A_55_P1987146 | Adgrf4 | -0,866811238 | A_55_P2076772 | Hspa5 | 0,40510661 |
| A_55_P2745205 | Gabrb2 | -0,858055773 | A_52_P209172 | Mrpl41 | 0,40738644 |
| A_55_P2399499 | B930025P03Rik | -0,855747526 | A_52_P681456 | Rftn2 | 0,4076501 |
| A_66_P112004 | LOC108167931 | -0,85463768 | A_55_P2086810 | Sp140 | 0,40777584 |
| A_66_P102265 | 1700119I11Rik | -0,851345322 | A_52_P237792 | Slc19a1 | 0,40786044 |
| A_55_P2006008 | Serpinb1a | -0,84694077 | A_55_P2796612 | Bag3 | 0,40800482 |
| A_66_P111765 | 1700026J14Rik | -0,842844066 | A_55_P2831026 | Sash3 | 0,40813752 |
| A_55_P2167323 | Cort | -0,836484546 | A_55_P1965772 | Atp2a3 | 0,40978326 |
| A_55_P2121729 | Gm35911 | -0,835892063 | A_55_P2099881 | Gm10461 | 0,41332425 |
| A_55_P1991039 | Gm7827 | -0,834527999 | A_66_P115844 | P2rx1 | 0,41432611 |
| A_66_P138779 | Gm16793 | -0,83220005 | A_55_P2022812 | Cyp4f17 | 0,41512001 |
| A_55_P2152607 | Cyp4a12b | -0,823442779 | A_52_P207509 | Peak1 | 0,41624303 |
| A_55_P2863646 | Gm32394 | -0,823029356 | A_51_P171999 | Apoe | 0,4164094 |
| A_55_P2139809 | Fgf13 | -0,822939104 | A_55_P2092750 | Car9 | 0,4171297 |
| A_66_P108216 | Klhl40 | -0,815095712 | A_51_P294020 | Ticam1 | 0,41806238 |
| A_55_P1958165 | Ms4a7 | -0,814075655 | A_55_P2803608 | Cpa4 | 0,41883108 |
| A_66_P120992 | Rnf152 | -0,812955986 | A_52_P302345 | Cyp4v3 | 0,42279263 |
| A_55_P2589772 | Tbc1d9 | -0,809645925 | A_55_P2883270 | Lpin1 | 0,42365862 |
| A_52_P82701 | Slc9a3 | -0,801430675 | A_51_P484020 | Cd300c2 | 0,42439283 |
| A_66_P107192 | Dhrs2 | -0,799584635 | A_55_P2125564 | Lrrc58 | 0,42452179 |
| A_51_P394515 | Tkt | -0,798688759 | A_51_P424054 | Btbd2 | 0,42471117 |
| A_51_P140742 | Islr | -0,798578357 | A_51_P117477 | Slc27a1 | 0,4256909 |
| A_55_P2715565 | Mdga2 | -0,798393744 | A_51_P177984 | Fam234a | 0,42619798 |
| A_52_P493477 | Serpinb1c | -0,794312888 | A_55_P2798054 | Rhoh | 0,42809676 |
| A_51_P337195 | Pipox | -0,793721424 | A_66_P111700 | Kyat3 | 0,42874219 |
| A_55_P2723949 | Gm1043 | -0,792418222 | A_51_P265151 | Arhgef10 | 0,43016019 |
| A_51_P516870 | Itm2a | -0,788081425 | A_55_P1968895 | Prph | 0,43064389 |
| A_51_P324351 | Meltf | -0,783864998 | A_55_P2743421 | Slc35d2 | 0,43377283 |
| A_66_P132695 | 1700015F17Rik | -0,782777418 | A_55_P2044932 | Gpr84 | 0,43447327 |
| A_66_P103848 | Gm34883 | -0,782614044 | A_51_P466148 | Bckdha | 0,43495876 |
| A_51_P470851 | Cplx3 | -0,777009953 | A_65_P17741 | Nxn | 0,43559382 |
| A_55_P2264231 | B230334L07Rik | -0,774652064 | A_52_P5579 | 4930455C13Rik | 0,43595167 |
| A_51_P386899 | Mfsd7c | -0,772026038 | A_55_P2092296 | Fbxo2 | 0,43816767 |
| A_55_P1998456 | Birc7 | -0,769575365 | A_55_P2820752 | Rhob | 0,43852699 |
| A_52_P556908 | Dlx6 | -0,767132627 | A_51_P452779 | Pygl | 0,44036406 |
| A_55_P2047639 | Cubn | -0,766562552 | A_55_P2131920 | Prrx1 | 0,44061202 |
| A_66_P116131 | Gm19500 | -0,764951517 | A_66_P137605 | Mb21d1 | 0,44092981 |
| A_52_P520037 | Rimbp2 | -0,764767192 | A_55_P1957173 | D14Ertd670e | 0,44121699 |
| A_55_P1968679 | Anks1b | -0,762049983 | A_55_P2077048 | Itih5 | 0,44142022 |
| A_51_P396193 | C4bp-ps1 | -0,761820931 | A_66_P131403 | Gm9465 | 0,44227821 |
| A_51_P193832 | Rtkn2 | -0,761820044 | A_55_P2931478 | Rxra | 0,44363538 |
| A_55_P1981814 | Myo3b | -0,761253252 | A_51_P431737 | Cth | 0,44547449 |
| A_52_P265051 | Sertm1 | -0,757742564 | A_51_P430929 | Fam20a | 0,44571717 |
| A_52_P249733 | Tcap | -0,752168323 | A_55_P2097508 | Mcc | 0,44604795 |
| A_55_P2499253 | Rsg1 | -0,752157094 | A_55_P2020371 | Gm11543 | 0,44677521 |
| A_66_P125576 | Gm30157 | -0,75209665 | A_51_P346668 | Irf5 | 0,44731579 |
| A_55_P2116993 | Hnrnpdl | -0,749008714 | A_55_P2112170 | Crtc1 | 0,44869901 |
| A_55_P2003638 | Stxbp6 | -0,745202531 | A_66_P135993 | Apobec3 | 0,44870119 |
| A_51_P501248 | Sphk1 | -0,739287547 | A_55_P2489870 | Emc8 | 0,45029277 |
| A_51_P428297 | 1700084M14Rik | -0,738056087 | A_51_P413348 | Ezr | 0,45233556 |
| A_51_P291417 | Thbd | -0,737506025 | A_66_P119594 | LOC102637491 | 0,45234779 |
| A_55_P2716881 | Brinp3 | -0,737497198 | A_52_P168567 | Cebpa | 0,45403452 |
| A_66_P114488 | Etaa1os | -0,735835385 | A_51_P451574 | Acot1 | 0,45685132 |
| A_66_P121636 | Ablim3 | -0,732560381 | A_55_P2139473 | Ilk | 0,45719262 |
| A_55_P2426564 | 1500004A13Rik | -0,732480207 | A_66_P139252 | Epb41l4aos | 0,45785851 |
| A_55_P2944462 | Ccdc116 | -0,731818684 | A_55_P2142222 | Serpina3h | 0,45885809 |
| A_51_P227077 | Mdh1b | -0,731054573 | A_55_P2803234 | Mtap | 0,459311 |
| A_51_P199778 | 9330171B17Rik | -0,73074401 | A_55_P2728573 | Adamts7 | 0,46123901 |
| A_55_P2759752 | A430010J10Rik | -0,729369644 | A_55_P2736989 | Tns2 | 0,46145631 |
| A_52_P119039 | Hmgcs1 | -0,728038859 | A_55_P2064771 | Ly6c1 | 0,4615145 |
| A_55_P2364211 | 2810428J06Rik | -0,726108058 | A_51_P327796 | Itgb5 | 0,46183563 |
| A_55_P2716117 | Il2ra | -0,723080746 | A_55_P2087539 | Clpsl2 | 0,46268593 |
| A_51_P473646 | Spx | -0,722850228 | A_55_P1954436 | Gm7967 | 0,46362205 |
| A_55_P2125376 | Cacna1d | -0,721621335 | A_55_P2161450 | Serpina3b | 0,46497351 |
| A_55_P2725610 | Gm12500 | -0,720650778 | A_55_P1975560 | Ifi204 | 0,46638344 |
| A_55_P2822536 | Apcdd1 | -0,718177789 | A_51_P167292 | Chil3 | 0,46702598 |
| A_66_P138013 | Snhg14 | -0,717635618 | A_51_P189746 | Pim3 | 0,46722772 |
| A_55_P2371105 | Sox2ot | -0,717577227 | A_55_P2067438 | Def8 | 0,46982067 |
| A_55_P2095301 | LOC105243282 | -0,713594019 | A_52_P49391 | Cyp2d22 | 0,47033851 |
| A_51_P244879 | AB041806 | -0,712689799 | A_66_P133112 | Gm14635 | 0,47138536 |
| A_51_P162162 | Inmt | -0,706576842 | A_55_P2732276 | Kif23 | 0,47148513 |
| A_55_P2566166 | Gm31137 | -0,706375827 | A_51_P100174 | Mns1 | 0,4722582 |
| A_51_P101985 | Ccdc85a | -0,705838381 | A_51_P417257 | Ppp1r2 | 0,47293369 |
| A_55_P2715321 | Cnksr2 | -0,700112226 | A_55_P2419116 | Snhg9 | 0,47302482 |
| A_52_P634090 | Jag1 | -0,696907185 | A_51_P167263 | Cd5 | 0,47471503 |
| A_55_P2796375 | Mycn | -0,693676306 | A_51_P290191 | Mst1 | 0,47561141 |
| A_55_P2288285 | Klhl33 | -0,69337173 | A_52_P177324 | Dlk1 | 0,477364 |
| A_66_P126235 | Gm26812 | -0,692925837 | A_65_P15133 | Apaf1 | 0,4780055 |
| A_51_P338878 | P2ry12 | -0,691316322 | A_51_P247359 | Ptprcap | 0,47895133 |
| A_51_P241068 | Dkk2 | -0,687246636 | A_55_P2046812 | Sparc | 0,47976575 |
| A_66_P100726 | C530044C16Rik | -0,686397451 | A_51_P288876 | Tmem45a | 0,48058613 |
| A_52_P157704 | Hs3st2 | -0,685519031 | A_55_P2722175 | Slc15a4 | 0,48087439 |
| A_55_P2806920 | Gabra5 | -0,684339287 | A_51_P473229 | Zbtb7b | 0,48122816 |
| A_52_P478174 | Sv2b | -0,68376166 | A_55_P2808254 | Frmpd1 | 0,48193381 |
| A_55_P2334927 | 9130022E09 | -0,67881844 | A_52_P495869 | Mafb | 0,48373944 |
| A_55_P2798408 | Alad | -0,677672649 | A_55_P2106844 | Gm35287 | 0,48431603 |
| A_55_P2162229 | LOC432842 | -0,677075021 | A_55_P2374337 | A130071D04Rik | 0,48465064 |
| A_51_P187226 | 1500015L24Rik | -0,676788677 | A_55_P2178800 | Ugt1a10 | 0,48549974 |
| A_55_P1978241 | Artn | -0,676690282 | A_55_P2002351 | Lats2 | 0,48590313 |
| A_51_P180492 | Dbp | -0,673416585 | A_51_P353232 | Tnnc2 | 0,48750413 |
| A_55_P2071691 | Kank3 | -0,670097869 | A_66_P110891 | Sgo2a | 0,487964 |
| A_52_P537907 | Tsga10 | -0,667622501 | A_55_P2410240 | Gm20556 | 0,48799488 |
| A_52_P228899 | Scn3b | -0,666963192 | A_51_P423356 | Katnal2 | 0,48807511 |
| A_55_P2800200 | 6430571L13Rik | -0,665558576 | A_51_P297679 | Hcls1 | 0,48874658 |
| A_55_P2012011 | Tceal7 | -0,664593397 | A_51_P391159 | Ang | 0,48963366 |
| A_55_P2808872 | Gldc | -0,663606295 | A_52_P502717 | Dpep2 | 0,48965215 |
| A_52_P635598 | Rgs8 | -0,660466875 | A_52_P502577 | S1pr3 | 0,490306 |
| A_66_P139554 | Pcsk2os2 | -0,657615352 | A_55_P2723654 | Lgals6 | 0,49050381 |
| A_51_P219594 | 1700123K08Rik | -0,655402826 | A_55_P2744012 | Exd1 | 0,4907093 |
| A_52_P518922 | Itga1 | -0,654459967 | A_55_P2140863 | Vmn1r124 | 0,49181772 |
| A_55_P2393021 | A430106G13Rik | -0,652981583 | A_55_P2912004 | Hmgcs2 | 0,49350423 |
| A_66_P128409 | A930024N18Rik | -0,652694234 | A_52_P212473 | Rnf125 | 0,49412883 |
| A_51_P228706 | Kcnc4 | -0,65105694 | A_51_P176352 | Ndrg2 | 0,49749763 |
| A_52_P467675 | Ankrd63 | -0,650852588 | A_52_P545613 | Fcgr2b | 0,49840232 |
| A_51_P231597 | Nkain3 | -0,649909881 | A_52_P656790 | Ctf2 | 0,49991658 |
| A_52_P580533 | Spata33 | -0,649857716 | A_52_P238953 | Dennd1b | 0,50042103 |
| A_66_P125352 | B230303O12Rik | -0,649548015 | A_51_P262208 | Itgb2 | 0,50151593 |
| A_51_P450365 | Txnrd3 | -0,646937535 | A_55_P2035326 | D5Ertd605e | 0,50301364 |
| A_55_P2028961 | Idi1 | -0,646412839 | A_51_P233396 | Ppp2r5d | 0,50498246 |
| A_51_P394833 | Tshz1 | -0,645994709 | A_55_P2831021 | Selplg | 0,50593004 |
| A_51_P209327 | Apln | -0,64516321 | A_55_P2066230 | Hck | 0,50820186 |
| A_55_P2177910 | Lepr | -0,64221962 | A_55_P2184606 | Itgal | 0,50861351 |
| A_52_P1051779 | Stum | -0,635036703 | A_51_P137150 | Dhx8 | 0,51038753 |
| A_65_P12774 | Adamts18 | -0,634766581 | A_55_P2765840 | Gm31645 | 0,51072282 |
| A_52_P318354 | Olfr1441 | -0,629849781 | A_51_P135423 | Capzb | 0,51119214 |
| A_55_P2508524 | B230216N24Rik | -0,629187075 | A_52_P637282 | Wipf1 | 0,51159016 |
| A_55_P2572127 | Gm36841 | -0,6285123 | A_66_P111992 | Gm36177 | 0,51174589 |
| A_66_P133553 | Cep126 | -0,62708125 | A_51_P197528 | Ly6c2 | 0,51259896 |
| A_55_P2049801 | Gm10629 | -0,625608976 | A_51_P235687 | Alox5ap | 0,51274723 |
| A_66_P107014 | Fam135b | -0,624742095 | A_55_P1999364 | Krt85 | 0,51304686 |
| A_66_P110662 | Ptn | -0,623625152 | A_55_P2408112 | 4930431P03Rik | 0,51449219 |
| A_52_P125253 | Olfr123 | -0,621943721 | A_55_P2568502 | Gm36254 | 0,51452155 |
| A_51_P230324 | Efcab3 | -0,620669333 | A_51_P208361 | Ak3 | 0,51463007 |
| A_51_P476018 | Sox7 | -0,617207347 | A_52_P671700 | Acad12 | 0,51578841 |
| A_55_P2718015 | Ube2f | -0,616801797 | A_55_P2100928 | Ptgds | 0,51633379 |
| A_51_P160463 | A230065N10Rik | -0,613726733 | A_55_P1984243 | Naip2 | 0,51862887 |
| A_66_P118891 | Mcpt-ps1 | -0,613580569 | A_52_P634250 | Lgals4 | 0,51890259 |
| A_55_P2582888 | Gm30609 | -0,611961034 | A_51_P279437 | Mfsd2a | 0,52092452 |
| A_52_P674808 | Chrdl1 | -0,608675887 | A_55_P2021119 | Sun2 | 0,52115874 |
| A_52_P589391 | Doc2a | -0,607959716 | A_52_P527500 | Helb | 0,5226101 |
| A_52_P480088 | Col27a1 | -0,603410766 | A_52_P511269 | Mlec | 0,52299616 |
| A_66_P109431 | Tmem125 | -0,599476678 | A_51_P327121 | Was | 0,52631598 |
| A_51_P117165 | 6330409D20Rik | -0,598301083 | A_55_P1973352 | Gm42035 | 0,52640963 |
| A_66_P136064 | Tmem132d | -0,596277127 | A_55_P2714414 | Sat1 | 0,52654768 |
| A_51_P418375 | Jam2 | -0,59295861 | A_51_P464387 | Hspb8 | 0,52790185 |
| A_55_P2914233 | Trim36 | -0,590525342 | A_52_P117393 | Tlr6 | 0,52815684 |
| A_55_P2015182 | Glt1d1 | -0,589523499 | A_51_P371001 | Tm4sf4 | 0,52938944 |
| A_55_P2117699 | Pak7 | -0,588427902 | A_55_P1961014 | Selenbp1 | 0,52975576 |
| A_55_P2022211 | Plxdc2 | -0,588309907 | A_51_P330580 | Mef2b | 0,52979491 |
| A_55_P2179572 | Slc18a3 | -0,587346367 | A_55_P1981789 | Rrp8 | 0,52999784 |
| A_52_P507498 | Plxnc1 | -0,585278799 | A_51_P338485 | Aldh6a1 | 0,53049981 |
| A_55_P2036788 | Zfhx2os | -0,58357477 | A_55_P1973906 | Trp53inp1 | 0,53326544 |
| A_51_P407480 | Myt1 | -0,581572099 | A_55_P2032718 | Klra9 | 0,53348562 |
| A_51_P261713 | Slco1c1 | -0,581292136 | A_51_P366138 | Mertk | 0,53637729 |
| A_55_P2373987 | A730009E18Rik | -0,580651724 | A_55_P2149122 | Farp2 | 0,53707015 |
| A_55_P2106434 | Zfp114 | -0,578272749 | A_55_P2792582 | Gm30713 | 0,53720766 |
| A_66_P124669 | A730020E08Rik | -0,572106362 | A_66_P102260 | Irs2 | 0,54004848 |
| A_55_P2107367 | Pbx3 | -0,568360856 | A_66_P131227 | Tcstv3 | 0,54256388 |
| A_52_P145349 | Stradb | -0,568286455 | A_55_P2070331 | Mirt1 | 0,5431616 |
| A_66_P109457 | 2410021H03Rik | -0,567476612 | A_55_P2148288 | Tmem79 | 0,54523739 |
| A_55_P2726960 | 1500035N22Rik | -0,562967868 | A_52_P244803 | D630033O11Rik | 0,54533185 |
| A_51_P335569 | Slco1a4 | -0,560451624 | A_55_P2216561 | 2500002B13Rik | 0,54632111 |
| A_66_P106235 | Lca5l | -0,557764981 | A_66_P131361 | Txnip | 0,54875257 |
| A_52_P523368 | Psapl1 | -0,55472221 | A_51_P220681 | Aldoc | 0,55082044 |
| A_65_P06678 | Msmo1 | -0,554251052 | A_52_P513177 | Sla | 0,55103806 |
| A_55_P1994418 | Teddm3 | -0,553483117 | A_52_P598309 | Zfas1 | 0,55362834 |
| A_51_P290074 | Fabp7 | -0,55335194 | A_51_P176365 | Gimap5 | 0,55400846 |
| A_55_P1966977 | Sntb1 | -0,550141994 | A_52_P220879 | Tgm2 | 0,55549141 |
| A_55_P2721832 | Sorbs2 | -0,549674397 | A_55_P2160825 | Xdh | 0,5560628 |
| A_55_P2503081 | Tbx18 | -0,545870926 | A_51_P237383 | Rnase4 | 0,55764271 |
| A_66_P109817 | Nova2 | -0,545829981 | A_51_P439403 | Padi1 | 0,55773701 |
| A_55_P2413722 | C130090I23Rik | -0,545614453 | A_55_P2169234 | Pycr1 | 0,55785988 |
| A_51_P412914 | Efs | -0,545135231 | A_51_P265495 | Ly6a | 0,55864322 |
| A_55_P2003541 | Nrcam | -0,545023363 | A_66_P139985 | 6030466F02Rik | 0,55880604 |
| A_51_P282609 | Grik1 | -0,544770456 | A_55_P2806054 | Olfml2b | 0,55891514 |
| A_55_P2908408 | Ppic | -0,542310514 | A_55_P2214625 | 4930519A11Rik | 0,55906118 |
| A_51_P265465 | Abhd3 | -0,54106506 | A_51_P216593 | Wfs1 | 0,5598031 |
| A_51_P477540 | Lyzl4 | -0,540299041 | A_51_P234044 | 1190005I06Rik | 0,55999834 |
| A_51_P292736 | Cd72 | -0,540188125 | A_55_P2244112 | Amotl1 | 0,56007612 |
| A_55_P2065288 | Scn3a | -0,537505173 | A_55_P2005320 | Dcstamp | 0,5600952 |
| A_51_P169745 | Tuba1a | -0,536319624 | A_52_P485007 | Abca2 | 0,560647 |
| A_55_P2713390 | Sema3c | -0,534510116 | A_51_P185660 | Ccl9 | 0,56121855 |
| A_65_P08480 | Abhd2 | -0,534421607 | A_55_P2010312 | Serpina1a | 0,56594039 |
| A_55_P2123162 | Prr5l | -0,532260913 | A_51_P106538 | Htra3 | 0,56654572 |
| A_55_P2883295 | Rcor2 | -0,529249262 | A_55_P2787236 | Gm32885 | 0,56899697 |
| A_55_P1981155 | Phldb1 | -0,526718754 | A_51_P147274 | Clec4a3 | 0,57077553 |
| A_55_P1961335 | Ctsk | -0,524845108 | A_51_P423578 | Slfn2 | 0,57175195 |
| A_55_P2778788 | Gm6556 | -0,519376407 | A_55_P2736690 | Dennd4c | 0,57404749 |
| A_51_P510418 | Aldh1b1 | -0,518532035 | A_66_P116502 | Il17rc | 0,57478878 |
| A_55_P2008417 | Mnd1 | -0,516785423 | A_55_P2022231 | Gm826 | 0,57592311 |
| A_55_P2721008 | Strada | -0,514099946 | A_66_P115451 | Gm5122 | 0,57702035 |
| A_55_P1958350 | Nipsnap3b | -0,513534132 | A_52_P615401 | Tpra1 | 0,57953815 |
| A_55_P2732021 | Tbx3 | -0,513494469 | A_52_P554536 | Tnfrsf26 | 0,57988944 |
| A_66_P123955 | Gm15704 | -0,510873074 | A_55_P1987730 | Fam213a | 0,58080933 |
| A_51_P334104 | Dcn | -0,508475387 | A_51_P167360 | Ptpn7 | 0,58320206 |
| A_66_P138470 | Gm17116 | -0,504305839 | A_51_P431020 | Selenbp2 | 0,58353523 |
| A_51_P383014 | Arhgap26 | -0,502616808 | A_51_P223404 | Plin3 | 0,58359524 |
| A_55_P2905383 | Rab33a | -0,500117963 | A_51_P190886 | Ankrd13a | 0,58397058 |
| A_55_P2059765 | Foxf1 | -0,49620559 | A_55_P1978191 | C2cd4c | 0,58483117 |
| A_55_P2045007 | Hrh1 | -0,494075831 | A_51_P503883 | Plekhg4 | 0,58510445 |
| A_55_P2732096 | Chl1 | -0,49401156 | A_51_P418725 | Plekhf1 | 0,58619634 |
| A_51_P238722 | Cd93 | -0,493772051 | A_55_P2062054 | Dbndd2 | 0,58692274 |
| A_66_P114206 | Tekt5 | -0,493458506 | A_55_P2106106 | C5ar2 | 0,58828302 |
| A_55_P2155226 | Ksr2 | -0,49174354 | A_55_P2853032 | Cp | 0,5886201 |
| A_51_P273667 | Sox17 | -0,490092825 | A_51_P255699 | Mmp3 | 0,58968819 |
| A_55_P2739535 | Prmt1 | -0,487012107 | A_55_P2119145 | Gm36913 | 0,59160061 |
| A_52_P566963 | Msi1 | -0,486764252 | A_55_P2717922 | Fyb | 0,59182758 |
| A_66_P104483 | Ciart | -0,483636132 | A_66_P121171 | Gm15413 | 0,59222736 |
| A_66_P135458 | Tmem108 | -0,482436533 | A_55_P2810149 | Gm20752 | 0,59249708 |
| A_51_P500541 | Flywch2 | -0,481165582 | A_66_P131467 | Mfap4 | 0,59360016 |
| A_55_P2335903 | Nlgn3 | -0,480422079 | A_51_P132013 | Cysltr2 | 0,59626531 |
| A_55_P2055587 | Enpp4 | -0,479779366 | A_51_P275496 | Gm39749 | 0,59635753 |
| A_55_P1992555 | Gys2 | -0,475907592 | A_66_P114811 | Pon2 | 0,597541 |
| A_52_P117352 | Gja4 | -0,47531924 | A_51_P345344 | Upk3bl | 0,59953079 |
| A_52_P553316 | Snrk | -0,474364505 | A_52_P299771 | Bcl2a1c | 0,60229524 |
| A_55_P1988388 | Gm2347 | -0,473530279 | A_51_P130115 | Spink2 | 0,60375042 |
| A_55_P2502396 | Epm2a | -0,472594393 | A_55_P1956160 | Gm8909 | 0,60445212 |
| A_52_P627327 | Nav3 | -0,465200387 | A_52_P49601 | Fth1 | 0,60875051 |
| A_55_P2928462 | Eme2 | -0,462603801 | A_51_P227392 | Rhou | 0,61605425 |
| A_55_P1966804 | Fdps | -0,462554785 | A_51_P470328 | Selenop | 0,61971987 |
| A_55_P1987434 | Fam196a | -0,45854862 | A_55_P2038262 | Phf11b | 0,62078661 |
| A_55_P2072233 | Zcchc12 | -0,456120955 | A_66_P121818 | Phc3 | 0,62095206 |
| A_51_P430082 | Tst | -0,454448931 | A_55_P2735715 | Pdgfra | 0,62392737 |
| A_55_P1961928 | Erich6 | -0,453844156 | A_51_P360004 | Ocstamp | 0,6253504 |
| A_55_P2125252 | Celf2 | -0,453066073 | A_52_P162695 | Pvr | 0,62575124 |
| A_52_P209484 | Tmem88 | -0,448884843 | A_55_P1994190 | Heph | 0,62645087 |
| A_51_P172502 | Cxcl12 | -0,448523565 | A_51_P331507 | Idh2 | 0,62761295 |
| A_55_P2241488 | 5330439K02Rik | -0,447208086 | A_55_P2545303 | Gm32191 | 0,63049534 |
| A_55_P2732718 | Fus | -0,447198518 | A_51_P357735 | Inhbe | 0,63220813 |
| A_52_P646515 | Clnk | -0,441319154 | A_66_P122086 | 9030619P08Rik | 0,64050546 |
| A_52_P84037 | Socs2 | -0,43759851 | A_55_P2091942 | BC061237 | 0,64051942 |
| A_65_P12160 | 2310039F13Rik | -0,437278511 | A_55_P2144736 | Prelp | 0,64347092 |
| A_55_P2729115 | Sema4b | -0,43642562 | A_55_P2910661 | Fgr | 0,64817419 |
| A_51_P284426 | Cstad | -0,435955552 | A_51_P114616 | Batf | 0,6491327 |
| A_51_P357043 | Slc26a10 | -0,435434905 | A_52_P129672 | Galm | 0,65320276 |
| A_51_P305547 | Snai2 | -0,431263607 | A_52_P23674 | Adgrf3 | 0,65371865 |
| A_55_P2507959 | Stmn1-rs1 | -0,429825924 | A_55_P2739022 | Acot11 | 0,6537442 |
| A_55_P2044385 | Fgfbp3 | -0,429342627 | A_52_P357829 | Gli2 | 0,65557571 |
| A_55_P2085075 | Gsx1 | -0,426946274 | A_66_P107608 | 6030443J06Rik | 0,65591961 |
| A_66_P132613 | C230057M02Rik | -0,424224295 | A_51_P104710 | Sspo | 0,65672273 |
| A_55_P2501636 | Dnm3 | -0,423677008 | A_52_P90265 | Ucp2 | 0,65711741 |
| A_65_P11059 | Letm1 | -0,422095088 | A_55_P2744772 | Treml2 | 0,65870563 |
| A_55_P2412319 | A830052D11Rik | -0,420976055 | A_51_P374376 | Olfr1366 | 0,65906028 |
| A_55_P2133001 | Bhlhe41 | -0,419790195 | A_55_P2809145 | Gm13373 | 0,660575 |
| A_51_P294535 | Unc5b | -0,418721388 | A_51_P424272 | Mt4 | 0,66265801 |
| A_55_P1972948 | Celf5 | -0,417639756 | A_66_P111660 | Mt1 | 0,66426743 |
| A_55_P2060193 | Mcf2l | -0,41519474 | A_51_P440365 | Frrs1 | 0,66462772 |
| A_51_P349495 | Mboat1 | -0,415073328 | A_51_P334942 | Aldh1a1 | 0,66670007 |
| A_52_P217710 | Fzd6 | -0,413039195 | A_52_P434055 | Birc3 | 0,67237197 |
| A_66_P117417 | Nectin3 | -0,412831004 | A_55_P1962851 | Gpr179 | 0,6728239 |
| A_55_P2128526 | Pfn2 | -0,408526634 | A_55_P2062190 | Gstm1 | 0,67326763 |
| A_51_P419726 | Ptprs | -0,403970273 | A_55_P2741734 | Parp3 | 0,67368364 |
| A_52_P639402 | Kcnk3 | -0,403144455 | A_51_P165182 | Batf2 | 0,67430417 |
| A_55_P2602800 | Zfp346 | -0,401337835 | A_66_P118021 | Hspa1b | 0,67635816 |
| A_52_P69194 | Rimkla | -0,400586458 | A_52_P106259 | Egfr | 0,67902183 |
| A_55_P2074499 | Kcp | -0,399747805 | A_51_P454949 | Gstm3 | 0,68050218 |
| A_55_P2733132 | Cacna2d1 | -0,398815306 | A_51_P270904 | Plekhs1 | 0,68116188 |
| A_55_P2214003 | 4932439E07Rik | -0,398776708 | A_55_P2048607 | Hp | 0,68150712 |
| A_55_P2150044 | Cdkl2 | -0,398377207 | A_51_P282837 | St14 | 0,68187046 |
| A_55_P2058726 | Hap1 | -0,397020099 | A_51_P246317 | Mt2 | 0,68192714 |
| A_55_P2250296 | 6330566A10Rik | -0,396037581 | A_52_P533707 | Chrna1 | 0,68319733 |
| A_55_P2509970 | A730017L22Rik | -0,394641312 | A_55_P2831118 | Mmp8 | 0,69068377 |
| A_55_P1985351 | Slc35f2 | -0,393228678 | A_66_P105132 | Oas1g | 0,69279739 |
| A_55_P2647945 | Plekha1 | -0,390172834 | A_66_P105958 | Sec31b | 0,69291622 |
| A_52_P586821 | Gng4 | -0,389468331 | A_51_P517695 | Ly6f | 0,69449568 |
| A_51_P485791 | Cyp51 | -0,389002424 | A_55_P2113738 | Ubash3a | 0,69581545 |
| A_66_P118579 | 3300002A11Rik | -0,38609917 | A_55_P2732788 | Rmi2 | 0,69603723 |
| A_66_P132218 | Gm4430 | -0,383743242 | A_51_P163188 | Rin2 | 0,69674132 |
| A_51_P432180 | Slc16a6 | -0,380647656 | A_52_P150651 | Cog5 | 0,70211886 |
| A_55_P2230506 | 4931402H11Rik | -0,380139804 | A_51_P493886 | Gpt2 | 0,70253271 |
| A_55_P2163659 | Rspo3 | -0,38000499 | A_66_P127789 | Bdkrb2 | 0,70704396 |
| A_55_P2348409 | D430022A14Rik | -0,375879834 | A_51_P392687 | Vim | 0,70980866 |
| A_55_P2163493 | Tgm3 | -0,374450876 | A_66_P100197 | Gm34403 | 0,71140233 |
| A_55_P2275402 | 9330177L23Rik | -0,373008741 | A_55_P2388040 | 4731419I09Rik | 0,71391038 |
| A_66_P125925 | Cntn2 | -0,372429421 | A_51_P208240 | Tnfsf14 | 0,7163599 |
| A_55_P2088018 | Fhod3 | -0,371465461 | A_51_P497171 | Ly9 | 0,71779874 |
| A_51_P461429 | Cyp7b1 | -0,363687297 | A_55_P2057528 | Arl4d | 0,71864403 |
| A_55_P1979728 | Atf4 | -0,363332564 | A_55_P1999883 | Fthl17-ps1 | 0,71907216 |
| A_55_P2033507 | Ltbp4 | -0,360169647 | A_55_P2023021 | Gm6961 | 0,72179838 |
| A_52_P706060 | Mex3a | -0,359670005 | A_51_P108459 | Gpr65 | 0,72445126 |
| A_55_P2130930 | Nhp2 | -0,359150453 | A_52_P576222 | Ppl | 0,72455033 |
| A_51_P359262 | Sec11c | -0,358844986 | A_55_P1984344 | Rhox2h | 0,72674333 |
| A_66_P135435 | Gm9099 | -0,358300106 | A_55_P2507380 | Lmntd2 | 0,72847552 |
| A_66_P136102 | Lefty2 | -0,358100353 | A_51_P349281 | Nckap5l | 0,72858449 |
| A_51_P341177 | Ddah2 | -0,357254604 | A_66_P114495 | Gm32478 | 0,7305136 |
| A_55_P2419483 | 4732460I02Rik | -0,356769781 | A_55_P1964955 | Gm3716 | 0,73248657 |
| A_55_P2721244 | Mtch2 | -0,355981545 | A_55_P2739891 | Abhd4 | 0,73638912 |
| A_55_P2171785 | Dnmt3aos | -0,354071887 | A_55_P2485525 | Pxmp2 | 0,73780998 |
| A_66_P140748 | 9430083A17Rik | -0,351864859 | A_51_P221248 | Tspan32 | 0,74216911 |
| A_55_P2032633 | LOC101055907 | -0,349444588 | A_55_P2609358 | Ddx58 | 0,74309119 |
| A_55_P2105685 | Adrb3 | -0,347558397 | A_55_P2135149 | Lcn12 | 0,74464382 |
| A_55_P1989996 | Tcte1 | -0,347166659 | A_66_P102722 | Bsph1 | 0,74809354 |
| A_55_P2742176 | Zcchc14 | -0,344479268 | A_66_P126913 | Tppp3 | 0,75108632 |
| A_51_P173459 | Nim1k | -0,343390825 | A_51_P239203 | Mapk13 | 0,75364725 |
| A_55_P2018904 | Kbtbd6 | -0,341808493 | A_52_P527800 | Emilin2 | 0,75810129 |
| A_55_P1989846 | Tm6sf1 | -0,340487831 | A_55_P2137701 | Zfp981 | 0,75828418 |
| A_51_P427516 | Thsd1 | -0,339582663 | A_55_P2324976 | 5033406O09Rik | 0,75906554 |
| A_55_P2733222 | Il15 | -0,339519677 | A_51_P376299 | Mchr1 | 0,76480624 |
| A_51_P455946 | Rac3 | -0,337709468 | A_52_P20867 | Tfcp2l1 | 0,76793576 |
| A_55_P1958921 | Ankrd29 | -0,337311211 | A_55_P2093286 | Apod | 0,77053473 |
| A_55_P2729011 | Gnas | -0,336917234 | A_55_P1978424 | Bcl2a1d | 0,77087581 |
| A_55_P2730961 | Syn2 | -0,334983386 | A_51_P457481 | Sectm1a | 0,77622779 |
| A_55_P2727654 | Gbe1 | -0,333449014 | A_66_P101561 | Gm2176 | 0,78131136 |
| A_52_P163795 | Tubb5 | -0,332015404 | A_55_P2804638 | Cd14 | 0,78138404 |
| A_51_P245405 | Ppp3cc | -0,331070464 | A_55_P2074678 | Gm3831 | 0,78599968 |
| A_51_P482711 | Dhcr24 | -0,330480379 | A_55_P2154933 | Cd200r4 | 0,78689025 |
| A_55_P2486689 | Yes1 | -0,32270539 | A_51_P182311 | Clec2i | 0,78830288 |
| A_51_P257960 | Psma5 | -0,32266851 | A_55_P2042247 | Nlrp2 | 0,79245681 |
| A_65_P10953 | Eif4g3 | -0,320684751 | A_55_P2245920 | Lrrc8a | 0,79338665 |
| A_55_P2035662 | Aebp1 | -0,320627764 | A_55_P2718810 | Psat1 | 0,79563639 |
| A_52_P82247 | Cycs | -0,320430336 | A_51_P372826 | Smox | 0,79638907 |
| A_55_P2110998 | Gpr153 | -0,318853009 | A_66_P125660 | Nde1 | 0,79672008 |
| A_52_P387009 | Egln3 | -0,318076947 | A_55_P2071466 | Ncf1 | 0,79803598 |
| A_55_P2719349 | Ralb | -0,31807574 | A_51_P497985 | C2 | 0,79989957 |
| A_52_P65506 | Cxxc4 | -0,317671087 | A_51_P513682 | Nuf2 | 0,80093491 |
| A_55_P1996988 | Papd5 | -0,314457466 | A_55_P2724684 | Il9r | 0,81033797 |
| A_52_P609024 | Cenpw | -0,314030821 | A_51_P459108 | Insl6 | 0,81161932 |
| A_55_P2792770 | Coq10b | -0,312719052 | A_55_P2472735 | A530032D15Rik | 0,81270979 |
| A_66_P119660 | Gpr165 | -0,312003226 | A_55_P1975682 | Rhox2e | 0,81338821 |
| A_51_P205286 | Pcsk4 | -0,31193834 | A_51_P209736 | Atoh8 | 0,81549017 |
| A_51_P251357 | Ctps | -0,309450048 | A_55_P2091461 | Casp4 | 0,81582435 |
| A_55_P2505000 | Fggy | -0,307596576 | A_51_P225224 | Htra1 | 0,81897741 |
| A_55_P2803990 | Kctd7 | -0,305732247 | A_51_P181517 | Fcgr4 | 0,82359912 |
| A_55_P2009534 | Ccdc88a | -0,303677991 | A_55_P2117559 | Siglec15 | 0,82611528 |
| A_52_P175242 | Irs1 | -0,30330058 | A_55_P1993473 | Gm1993 | 0,82946682 |
| A_51_P258848 | Mfsd13b | -0,303059345 | A_51_P258372 | Igsf6 | 0,83725801 |
| A_55_P2723801 | Gm17066 | -0,302176444 | A_51_P115178 | Scara3 | 0,84356962 |
| A_51_P441451 | Foxred2 | -0,301625391 | A_51_P470353 | 4930412O13Rik | 0,84380958 |
| A_55_P1958867 | Higd1a | -0,30000667 | A_55_P2096630 | Ssxb9 | 0,84652781 |
| A_55_P2105858 | Atf5 | -0,297373401 | A_51_P131335 | Lrrc66 | 0,84865469 |
| A_55_P2117764 | Vangl2 | -0,29514255 | A_51_P420229 | Ccr7 | 0,86168686 |
| A_55_P1985882 | Fastkd1 | -0,29382861 | A_51_P335146 | H60a | 0,86407858 |
| A_55_P1992194 | Mboat7 | -0,293549347 | A_52_P638283 | Fam71f1 | 0,86611001 |
| A_51_P202033 | Wls | -0,293233261 | A_51_P341203 | Cyp3a41a | 0,87045518 |
| A_55_P2716944 | Cnrip1 | -0,290576962 | A_66_P101819 | E030042O20Rik | 0,8743637 |
| A_55_P2077783 | Tubb2a-ps2 | -0,290497897 | A_51_P489522 | Ctla2b | 0,87802894 |
| A_51_P107326 | Oxct1 | -0,289711383 | A_52_P265584 | 1810011O10Rik | 0,88027293 |
| A_55_P2908104 | Dck | -0,287607175 | A_51_P344018 | Klra12 | 0,88225256 |
| A_51_P172532 | Nit1 | -0,286471989 | A_66_P103894 | Zdhhc22 | 0,88380725 |
| A_52_P179178 | Bend3 | -0,285270854 | A_55_P2509543 | Gm14005 | 0,88873841 |
| A_55_P2118520 | Col1a1 | -0,285065191 | A_55_P1961039 | Pnpla2 | 0,89284832 |
| A_51_P343323 | Cox6c | -0,28498649 | A_51_P323248 | Sdc4 | 0,89342764 |
| A_55_P2129856 | Mtfmt | -0,284050634 | A_51_P179701 | Hlx | 0,89657517 |
| A_52_P883941 | Gm46304 | -0,283803182 | A_55_P2549836 | 2310003N18Rik | 0,89773922 |
| A_55_P1974467 | Ndufb6 | -0,283174601 | A_55_P1996893 | Zpbp2 | 0,8993144 |
| A_55_P2016959 | Nme2 | -0,282704682 | A_55_P2106039 | Map3k6 | 0,89972421 |
| A_52_P572447 | Agpat5 | -0,281701455 | A_55_P2046563 | Cym | 0,90621395 |
| A_52_P229728 | Cdc42 | -0,279191109 | A_51_P174961 | F10 | 0,91168675 |
| A_66_P118233 | Hnrnpa0 | -0,276584387 | A_51_P321341 | Sult1a1 | 0,91462217 |
| A_51_P424499 | Txn1 | -0,272572584 | A_51_P379807 | Cyp4f41-ps | 0,91676049 |
| A_51_P239673 | Hprt | -0,272456583 | A_51_P319070 | Retsat | 0,91882831 |
| A_55_P2783367 | Tmem14c | -0,269000397 | A_55_P2314852 | LOC102632594 | 0,92086095 |
| A_55_P2154228 | Ncapd3 | -0,267917343 | A_52_P459564 | Itga2b | 0,92559598 |
| A_55_P2912968 | Tuba3a | -0,265983847 | A_52_P408757 | Fcgr3 | 0,92717176 |
| A_52_P297773 | Uggt2 | -0,264613459 | A_66_P110617 | Men1 | 0,92783411 |
| A_52_P869802 | Thoc3 | -0,262415269 | A_66_P121526 | Kank4os | 0,94743 |
| A_66_P125317 | Helq | -0,260036117 | A_55_P2783103 | LOC735298 | 0,94902759 |
| A_52_P548470 | Shank2 | -0,256481907 | A_55_P2106800 | Bglap | 0,95205372 |
| A_51_P143805 | Tmem42 | -0,256338115 | A_55_P1955656 | Ctla2a | 0,96087013 |
| A_51_P216005 | Col4a5 | -0,255859386 | A_55_P2483227 | Cdca7 | 0,96263984 |
| A_51_P474454 | Rnaseh2b | -0,254835057 | A_55_P2076941 | Lrr1 | 0,96509222 |
| A_55_P2046398 | Eif4a1 | -0,253136764 | A_51_P137419 | Cst7 | 0,9666934 |
| A_52_P139747 | Ndufa1 | -0,252356591 | A_55_P2515994 | Rorc | 0,96730899 |
| A_51_P367843 | Polr2g | -0,250796774 | A_55_P1976204 | Cdkn1a | 0,97383878 |
| A_66_P102090 | Pkmyt1 | -0,249967476 | A_51_P191463 | H2-M2 | 0,97717503 |
| A_51_P172801 | Memo1 | -0,249648307 | A_55_P2157378 | Xlr5a | 0,9774368 |
| A_52_P421947 | Mtss1 | -0,249317678 | A_65_P08125 | D14Ertd426e | 0,98671378 |
| A_51_P200484 | Lrp12 | -0,248985204 | A_51_P294555 | Ifitm6 | 0,98689718 |
| A_51_P415945 | Psma2 | -0,248647811 | A_55_P1962305 | Plac8 | 0,98750094 |
| A_55_P2100118 | Nme1 | -0,246901622 | A_51_P254855 | Ptgs2 | 0,98793789 |
| A_55_P2721965 | Zfp30 | -0,246199605 | A_55_P2562770 | Gm30961 | 0,98983091 |
| A_55_P2847596 | Cops5 | -0,245879021 | A_55_P1982947 | Gm32742 | 0,99181171 |
| A_55_P2730208 | P4htm | -0,24494741 | A_51_P497882 | Creb3l4 | 0,99202802 |
| A_66_P103403 | Pdhb | -0,242229189 | A_66_P116477 | Selp | 0,9962908 |
| A_51_P329251 | Chchd1 | -0,241488061 | A_55_P2026567 | Ush2a | 1,00056582 |
| A_55_P2152049 | Xrcc3 | -0,240631857 | A_51_P233727 | Sapcd1 | 1,00768461 |
| A_66_P108468 | 2610528A11Rik | -0,236790386 | A_52_P608322 | Maff | 1,0092286 |
| A_55_P2016316 | Xrn2 | -0,235588292 | A_51_P319460 | Osmr | 1,01426832 |
| A_52_P81468 | Psma4 | -0,235253957 | A_52_P658320 | Mfsd7a | 1,02157385 |
| A_51_P411770 | Nip7 | -0,235044748 | A_52_P153929 | Brip1 | 1,02520249 |
| A_55_P2060253 | Gm12657 | -0,232215156 | A_55_P2108151 | Hbb-b1 | 1,02931998 |
| A_52_P370484 | Uqcrq | -0,231759326 | A_51_P183239 | Fancd2 | 1,03050123 |
| A_55_P2037116 | Ran | -0,228775978 | A_55_P2016882 | Nlrp4c | 1,04178877 |
| A_52_P560620 | Abhd11 | -0,228066204 | A_55_P2367350 | 4933403J19Rik | 1,04197856 |
| A_55_P2016682 | Alkbh7 | -0,227403184 | A_55_P2028734 | Klra16 | 1,04338694 |
| A_51_P292754 | Yaf2 | -0,226780574 | A_66_P111090 | Sctr | 1,04751993 |
| A_55_P2051874 | Psmc2 | -0,225747709 | A_55_P2284273 | A430085C19 | 1,05015952 |
| A_51_P137388 | Zadh2 | -0,224050426 | A_51_P302273 | Usp17la | 1,05076085 |
| A_55_P2154977 | Rpap3 | -0,223574785 | A_51_P349221 | Fcrl1 | 1,0520365 |
| A_51_P228632 | Rap1a | -0,21928521 | A_55_P1988985 | Vmn1r151 | 1,05713356 |
| A_55_P2924955 | Gm11423 | -0,218177236 | A_55_P1973809 | Hbb-bt | 1,06698915 |
| A_51_P509941 | Psmc6 | -0,2165091 | A_55_P2184567 | Gm3161 | 1,06799845 |
| A_55_P1953387 | Fabp5 | -0,215797637 | A_55_P2061485 | Mybl2 | 1,08515069 |
| A_51_P470242 | Rasl2-9 | -0,214753051 | A_66_P118807 | Gm38402 | 1,09336129 |
| A_51_P396752 | Arl2bp | -0,209940998 | A_55_P2026632 | Pilrb1 | 1,10746723 |
| A_51_P384879 | Mdh2 | -0,208119455 | A_66_P121976 | Slc4a11 | 1,11235592 |
| A_55_P2084631 | Hist1h2an | -0,204740804 | A_55_P2124043 | Tceanc | 1,11446401 |
| A_51_P243930 | Qrsl1 | -0,204637671 | A_55_P1962404 | Ugt1a6b | 1,11602544 |
| A_55_P2051962 | Sec61b | -0,20387937 | A_51_P276235 | Pnpla7 | 1,11838086 |
| A_55_P2068265 | Mif | -0,200782991 | A_66_P126848 | Fthl17f | 1,11994289 |
| A_51_P279127 | Fam173a | -0,199992966 | A_55_P2006035 | Galnt15 | 1,13637861 |
| A_51_P144770 | 2410002F23Rik | -0,198424333 | A_51_P247249 | Alox5 | 1,14473578 |
| A_66_P111675 | Rap1b | -0,196639552 | A_51_P126236 | Gm14461 | 1,15010341 |
| A_55_P2857230 | Bzw1 | -0,191264584 | A_51_P424854 | Ahsg | 1,1527546 |
| A_66_P129586 | Atp5o | -0,185698402 | A_51_P144632 | Sit1 | 1,16213196 |
| A_55_P2149719 | Atp5k | -0,183562416 | A_66_P117032 | C4b | 1,16410838 |
| A_51_P157595 | Snrpc | -0,180855793 | A_51_P386270 | Cyp2d12 | 1,16459888 |
| A_51_P164425 | Mrps16 | -0,179404811 | A_51_P517051 | Gatsl3 | 1,16869358 |
| A_55_P2148534 | Nr1d2 | -0,172990749 | A_52_P526740 | A430093F15Rik | 1,17279131 |
|  |  |  | A_51_P159453 | Serpina3n | 1,17447357 |
|  |  |  | A_52_P497134 | Dlx4os | 1,18490502 |
|  |  |  | A_51_P408506 | Icam1 | 1,19180545 |
|  |  |  | A_55_P2744479 | Phyhd1 | 1,19640749 |
|  |  |  | A_51_P231820 | C130026I21Rik | 1,19922031 |
|  |  |  | A_51_P350453 | Pdk4 | 1,20537464 |
|  |  |  | A_55_P2768796 | Gm35933 | 1,21250024 |
|  |  |  | A_55_P2031058 | Mc2r | 1,2172315 |
|  |  |  | A_55_P2048961 | Rbmy | 1,22014774 |
|  |  |  | A_51_P338443 | Angptl4 | 1,23921372 |
|  |  |  | A_52_P262930 | 2310081J21Rik | 1,24300411 |
|  |  |  | A_51_P377995 | Ptprq | 1,25917052 |
|  |  |  | A_66_P138866 | Apol7d | 1,26533131 |
|  |  |  | A_55_P2057577 | Ugt1a6a | 1,29582305 |
|  |  |  | A_55_P2025721 | Adam3 | 1,30448147 |
|  |  |  | A_55_P2053309 | Rgs21 | 1,3053795 |
|  |  |  | A_55_P2088237 | Cyp27b1 | 1,31144856 |
|  |  |  | A_66_P134542 | Anln | 1,32277413 |
|  |  |  | A_55_P2829197 | Gm32023 | 1,34305975 |
|  |  |  | A_51_P409250 | Oog1 | 1,35423795 |
|  |  |  | A_66_P125111 | Gm38483 | 1,35591858 |
|  |  |  | A_51_P143893 | Steap4 | 1,36760319 |
|  |  |  | A_55_P2508728 | Gm15471 | 1,37224746 |
|  |  |  | A_52_P593379 | Pax9 | 1,41179835 |
|  |  |  | A_55_P2745402 | C4a | 1,41332738 |
|  |  |  | A_51_P211616 | Slc27a6 | 1,41418813 |
|  |  |  | A_55_P2266013 | Gm13032 | 1,42473134 |
|  |  |  | A_55_P2040838 | Gm14548 | 1,42927419 |
|  |  |  | A_55_P1998471 | S100a9 | 1,43671618 |
|  |  |  | A_52_P627816 | Tgm1 | 1,43856532 |
|  |  |  | A_55_P2127699 | Pira2 | 1,45899647 |
|  |  |  | A_55_P1957922 | Arrdc2 | 1,46031627 |
|  |  |  | A_66_P129266 | Gm11538 | 1,46748486 |
|  |  |  | A_51_P499838 | Bst1 | 1,47001222 |
|  |  |  | A_55_P1962299 | Hba-a2 | 1,50584932 |
|  |  |  | A_55_P1962303 | Hba-a1 | 1,51430999 |
|  |  |  | A_52_P288050 | Fbln5 | 1,52025293 |
|  |  |  | A_66_P137447 | 2610017A05Rik | 1,52407591 |
|  |  |  | A_55_P2012071 | Sh2d1a | 1,52581328 |
|  |  |  | A_55_P2098408 | Olfr630 | 1,52582287 |
|  |  |  | A_52_P670026 | Rsad2 | 1,54192906 |
|  |  |  | A_51_P326191 | Serpina3g | 1,5483176 |
|  |  |  | A_55_P2034033 | Il12rb1 | 1,54886415 |
|  |  |  | A_51_P474029 | Rhox13 | 1,5538929 |
|  |  |  | A_51_P187602 | Serpinb5 | 1,56443198 |
|  |  |  | A_51_P444447 | Cebpd | 1,56974408 |
|  |  |  | A_55_P1998011 | Klra23 | 1,58015059 |
|  |  |  | A_55_P2072095 | Dpcr1 | 1,58978147 |
|  |  |  | A_55_P2272032 | 9130001E16Rik | 1,59033799 |
|  |  |  | A_55_P2716496 | Rhpn2 | 1,65603897 |
|  |  |  | A_51_P413866 | Cfb | 1,66540371 |
|  |  |  | A_66_P138201 | Rhox10 | 1,69968492 |
|  |  |  | A_51_P346938 | Lrg1 | 1,70777244 |
|  |  |  | A_52_P300451 | Tcf23 | 1,74283691 |
|  |  |  | A_51_P467076 | Cyp2b9 | 1,7489662 |
|  |  |  | A_52_P278274 | Olfr490 | 1,75307953 |
|  |  |  | A_55_P2051400 | Prl3d3 | 1,76419032 |
|  |  |  | A_51_P110341 | Scgb3a1 | 1,80338143 |
|  |  |  | A_55_P2104975 | Serpina3f | 1,80521421 |
|  |  |  | A_55_P2079579 | Pira7 | 1,82011189 |
|  |  |  | A_55_P2173768 | Pkhd1 | 1,82062658 |
|  |  |  | A_51_P173678 | Slc10a6 | 1,8569805 |
|  |  |  | A_51_P386870 | Sprr2f | 1,90868951 |
|  |  |  | A_55_P2725772 | Gm32695 | 1,96597201 |
|  |  |  | A_52_P1058797 | Gm26688 | 2,04070389 |
|  |  |  | A_55_P2046959 | Mmp27 | 2,06540081 |
|  |  |  | A_55_P2743391 | Apob | 2,07626982 |
|  |  |  | A_51_P434737 | BC023105 | 2,07999318 |
|  |  |  | A_55_P1971744 | Gm10057 | 2,10067013 |
|  |  |  | A_55_P2834410 | Gm33318 | 2,10430952 |
|  |  |  | A_66_P126679 | 1700080G11Rik | 2,11335974 |
|  |  |  | A_51_P161054 | Itgb6 | 2,13278707 |
|  |  |  | A_66_P103230 | LOC433354 | 2,15779945 |
|  |  |  | A_55_P2105643 | Mmp1b | 2,25097789 |
|  |  |  | A_51_P510156 | Lcn2 | 2,44602151 |
|  |  |  | A_66_P127692 | AV064505 | 2,48304217 |
|  |  |  | A_52_P60194 | C4bp | 2,5049914 |
|  |  |  | A_55_P2489334 | Gm3259 | 2,52807577 |
|  |  |  | A_55_P2142789 | AI463229 | 2,53821792 |
|  |  |  | A_52_P63680 | Acer1 | 2,67968644 |
|  |  |  | A_55_P2165929 | Olfr3 | 2,87300362 |
|  |  |  | A_52_P58359 | Tlx1 | 2,91735752 |
|  |  |  | A_55_P2075553 | Tnfsf18 | 2,98201518 |
|  |  |  | A_51_P112966 | Ch25h | 3,00661793 |
|  |  |  | A_51_P495780 | Plin4 | 3,09785683 |
|  |  |  | A_52_P42245 | Klrb1a | 3,24251461 |
|  |  |  | A_55_P2142941 | G370120E05Rik | 3,61046408 |
|  |  |  | A_51_P108226 | Wfdc21 | 3,99711717 |
|  |  |  | A_51_P256827 | S100a8 | 4,4672053 |


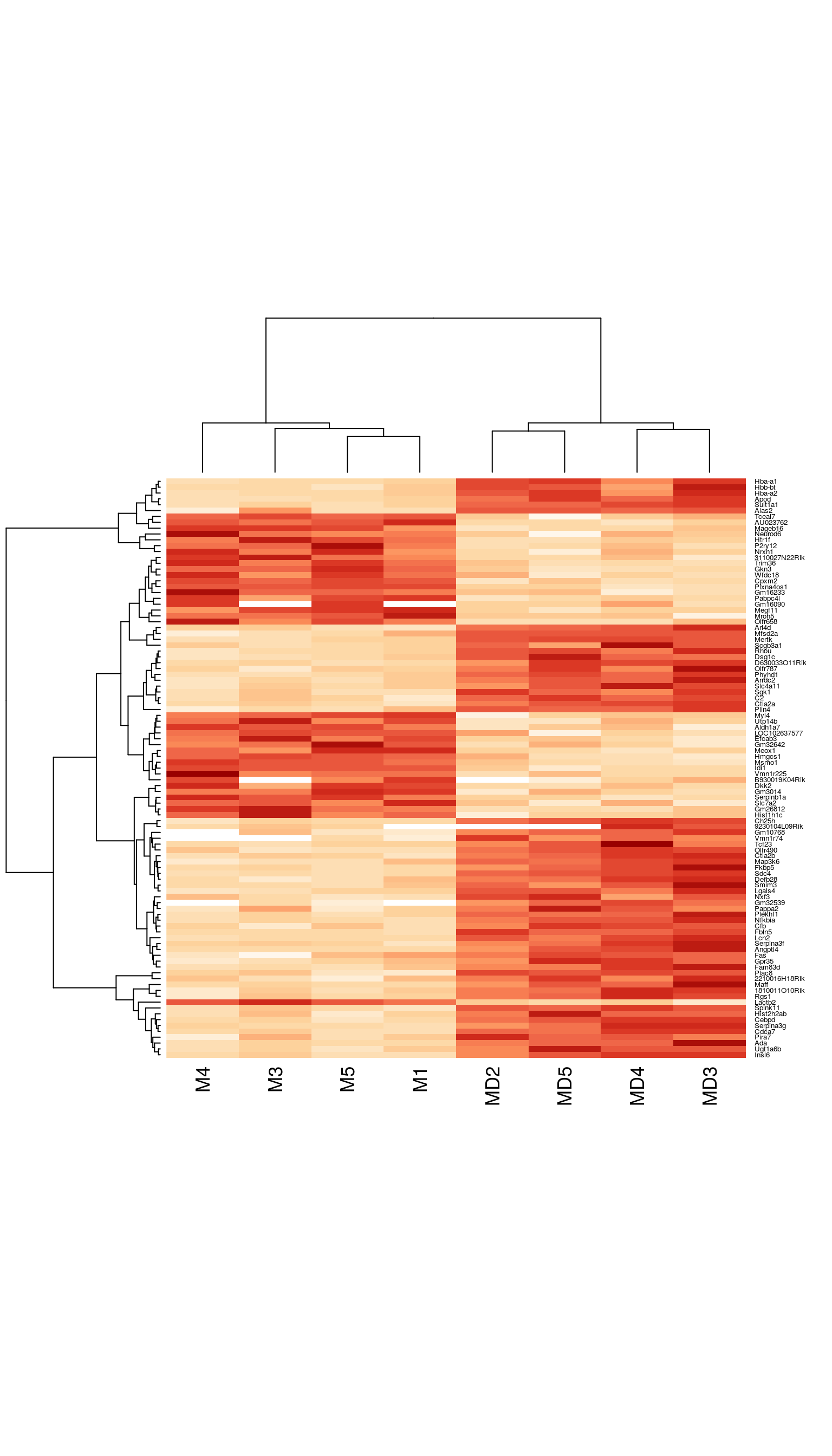


**Supplementary Figure 1.4.**: The cluster heat map of differentially expressed genes (DEGs) of individual samples from MPTP (M1-M4) and MPTP+DSS (MD1-MD4) animals. Horizontal axis stands for samples of microarray data and vertical axis stands for DEGs. The upper half stands for down-regulated DEGs; data column of Parkinson’s disease (PD) samples was mainly green and healthy control (HC) samples was mainly red. The lower half stands for up- regulated DEGs; data column of PD samples was mainly red and HC samples was mainly green PPI.
